# Pheno- and Genotyping of Hopanoid Production in *Acidobacteria*

**DOI:** 10.3389/fmicb.2017.00968

**Published:** 2017-06-08

**Authors:** Jaap S. Sinninghe Damsté, W. Irene C. Rijpstra, Svetlana N. Dedysh, Bärbel U. Foesel, Laura Villanueva

**Affiliations:** ^1^Department of Marine Microbiology and Biogeochemistry, NIOZ Royal Netherlands Institute for Sea Research, Utrecht UniversityDen Burg, Netherlands; ^2^Department of Earth Sciences, Geochemistry, Faculty of Geosciences, Utrecht UniversityUtrecht, Netherlands; ^3^S. N. Winogradsky Institute of Microbiology, Research Center of Biotechnology of Russian Academy of SciencesMoscow, Russia; ^4^Department of Microbial Ecology and Diversity Research, German Collection of Microorganisms and Cell Cultures (LG)Braunschweig, Germany

**Keywords:** Hopanoids, *Acidobacteria*, lipid biosynthesis, lipid analysis, methylation, genomes, metagenomes

## Abstract

Hopanoids are pentacyclic triterpenoid lipids synthesized by different bacterial groups. Methylated hopanoids were believed to be exclusively synthesized by cyanobacteria and aerobic methanotrophs until the genes encoding for the methylation at the C-2 and C-3 position (*hpn*P and *hpn*R) were found to be widespread in the bacterial domain, invalidating their use as specific biomarkers. These genes have been detected in the genome of the *Acidobacterium* “*Ca*. Koribacter versatilis,” but our knowledge of the synthesis of hopanoids and the presence of genes of their biosynthetic pathway in other member of the *Acidobacteria* is limited. We analyzed 38 different strains of seven *Acidobacteria* subdivisions (SDs 1, 3, 4, 6, 8, 10, and 23) for the presence of C_30_ hopenes and C_30+_ bacteriohopane polyols (BHPs) using the Rohmer reaction. BHPs and/or C_30_ hopenes were detected in all strains of SD1 and SD3 but not in SD4 (excepting *Chloracidobacterium thermophilum*), 6, 8, 10, and 23. This is in good agreement with the presence of genes required for hopanoid biosynthesis in the 31 available whole genomes of cultivated *Acidobacteria*. All genomes encode the enzymes involved in the non-mevalonate pathway ultimately leading to farnesyl diphosphate but only SD1 and 3 *Acidobacteria* and *C. thermophilum* encode all three enzymes required for the synthesis of squalene, its cyclization (*shc*), and addition and modification of the extended side chain (*hpnG, hpnH, hpnI, hpnJ, hpnO*). In almost all strains, only tetrafunctionalized BHPs were detected; three strains contained variable relative abundances (up to 45%) of pentafunctionalized BHPs. Only “*Ca*. K. versatilis” contained methylated hopanoids (i.e., 2,3-dimethyl bishomohopanol), although in low (<10%) amounts. These genes are not present in any other *Acidobacterium*, consistent with the absence of methylated BHPs in the other examined strains. These data are in agreement with the scattered occurrence of methylated BHPs in other bacterial phyla such as the *Alpha*-, *Beta*-, and *Gammaproteobacteria* and the *Cyanobacteria*, limiting their biomarker potential. Metagenomes of *Acidobacteria* were also examined for the presence of genes required for hopanoid biosynthesis. The complete pathway for BHP biosynthesis was evident in SD2 *Acidobacteria* and a group phylogenetically related to SD1 and SD3, in line with the limited occurrence of BHPs in acidobacterial cultures.

## Introduction

Hopanoids are omnipresent natural products occurring in many groups of bacteria and some higher plants. The recognition of their widespread occurrence in the bacterial domain was initiated by the ubiquitous presence of molecular fossils derived from hopanoids in the geological record (Ourisson et al., 1979). Hopanoids occur in a variety of structures from relatively simple C_30_ components to elongated compounds with a polyfunctionalized side chain, i.e., the bacteriohopanepolyols (BHPs), often containing additional substituents, i.e., the so-called BHP derivatives (see for reviews Kannenberg and Poralla, 1999 and Rezanka et al., 2010).

The large variety in structure and their ubiquitous presence in sediments and petroleum has resulted in the common application of hopanoids as molecular fossils. For example, the occurrence of hopanoids methylated at the C-2 position in the A-ring of hopanoids were thought to be limited to cyanobacteria. Consequently, their fossil occurrence has been used to reveal the timing of the advent of oxygenic photosynthesis (Summons et al., 1999). The abundance of 2-methyl extended hopanes relative to their non-methylated counterparts was used to conclude that nitrogen-fixing cyanobacteria played a key role in the deposition of black shales during the early Aptian and late Cenomanian oceanic anoxic events (Kuypers et al., 2004). Such studies strongly rely on the consistency of the link between cyanobacteria and their unique ability to produce 2-methyl BHPs. This link was already somewhat questionable since non-extended 2-methyl hopanoids (i.e., 2ß-methyldiplopterol and 2ß-methyldiploptene) were also reported in species falling in the group of the *Alphaproteobacteria* (Zundel and Rohmer, 1985; Knani et al., 1994; Vilcheze et al., 1994; Bravo et al., 2001). Subsequently, an extended 2-methyl hopanoid was detected in the anoxygenic phototroph, *Rhodopseudomonas palustris* (Rashby et al., 2007), also belonging to the *Alphaproteobacteria*. This revealed that the potential origins of sedimentary extended 2-methyl hopanoids are more diverse than previously thought.

This example highlights the importance of studies of lipids in cultured microbes for our interpretation of the molecular fossil record. However, such studies are laborious and have therefore been limited in number. Furthermore, they are fundamentally biased by the fact that the majority of the environmentally significant microbes are not available in culture. One approach to solve these issues is to use the genetic information of microbes that encodes the biochemical machinery of the cell, including their lipid biosynthetic pathways. Applying this approach in hopanoid research, Pearson et al. (2007, 2009) investigated the gene encoding the squalene-hopene cyclase (*shc*), which represents the first step in BHP biosynthesis, to search in the environment for potential producers of BHPs. Welander et al. (2010) also used a genetic approach and identified a radical SAM methylase encoded by a gene (*hpnP*) that is required for the methylation of hopanoids at the C-2 position. They demonstrated that this *hpnP* gene is not only present in cyanobacteria, but also in *Alphaproteobacteria* and *Acidobacteria*, casting further doubt on the application of 2-methyl BHPs as indicators for cyanobacteria. In a follow-up paper (Ricci et al., 2014) they used this gene to search for potential sources of 2-methyl hopanoids and reported environmental *hpnP* genes for cyanobacteria, alpha proteobacteria, but not for acidobacteria. Welander and Summons (2012) showed that a gene, *hpnR*, is required for methylation of hopanoids at the C-3 position in *Methylococcus capsulatus*. This gene is found in genomes of methanotrophic and acetic acid bacteria but also in other bacteria such as *Actinobacteria, Nitrospirae*, and *Acidobacteria* and, consequently, 3-methyl hopanoids cannot be used as biomarkers for aerobic methanotrophs.

*Acidobacteria* are a highly abundant and diverse phylum of the domain *Bacteria* (e.g., Dedysh et al., 2006; Janssen, 2006; Jones et al., 2009) and, therefore, could potentially be important sources for 2- and 3-methyl BHPs in the environment. The phylogenetic breadth of the *Acidobacteria* is comparable to that of the *Proteobacteria* (see Kielak et al., 2016 for a recent review). They have been divided in 26 subdivisions (SDs), mainly based on environmental sequences (Barns et al., 2007), but only seven of these contain taxonomically characterized representatives (Kielak et al., 2016). For SD1, eleven genera have been defined, and for SD4 eight genera, while a more limited number of genera (1–4 genera) have been characterized for SDs 3, 6, 8, 10, and 23. All cultured acidobacteria are heterotrophic and are able to grow on a variety of substrates. Molecular ecological studies have revealed that, in wetlands, the most abundant *Acidobacteria* fall in SD1 and 3 (Serkebaeva et al., 2013), whereas in lakes SDs 1, 6, and 7 thrive (Zimmermann et al., 2012). In soils, SDs 1–4 and 6 are most abundant (Jones et al., 2009). The hopanoids produced by *Acidobacteria* may thus form a major source of hopanoids in the environment. Since Welander et al. (2010) and Welander and Summons (2012) detected the *shc* gene and two genes involved in the methylation of hopanoids at the C-2 and the C-3 position in “*Candidatus* Koribacter versatilis,” an SD1 acidobacterium isolated from soil (Joseph et al., 2003) for which the whole genome is available (Ward et al., 2009), we cultivated “*Ca*. K. versatilis” and 37 other acidobacterial strains of SDs 1, 3, 4, 6, 8, 10, and 23 in order to test if *Acidobacteria* can indeed be an important source of specific hopanoids in the environment. We compared these results with the presence of genes involved in the biosynthetic pathway leading to BHPs.

## Materials and methods

### Cultures

The acidobacterial strains used in this study are listed in Table 1 and mostly were cultivated under conditions previously described (Sinninghe Damsté et al., 2011, 2014). “*Ca*. Koribacter versatilis Ellin345” (DSM 22529) and “*Ca*. Solibacter usitatus Ellin6076” (DSM 22595) were cultivated at 25°C in VL55 media, *Geothrix fermentans* (DSM 14018) was grown at 32°C in Geobacter medium (M 826, DSMZ media list) and *Holophaga foetida* (DSM 6591) at 28°C in TMBS4 medium (M 559 from DSMZ media list). The two species of SD6 *Acidobacteria* were grown as described earlier (Huber et al., 2016; Vieira et al., 2017).

**Table 1:** Presence of BHPs (both penta- and tetrafunctionalized in %) and hop-17(21)-ene in cultivated *Acidobacteria* of SDs 1, 3, 4, 6, 8, 10, and 23.

**Acidobacterium**	***SD***	**Origin**	**References**	**BHP**		**Hop-17(21)-ene**
				**Penta**	**Tetra**	
*Edaphobacter aggregans* Wbg-1^T^ (= DSM 19364^T^)	1	Alpine soil	Koch et al., 2008	–	100	–
*Edaphobacter modestus* Jbg-1^T^ (= DSM 18101^T^)	1	Forest soil	Koch et al., 2008	n.a.	n.a.	✓
*Acidobacterium capsulatum* 161^T^ (= DSM 11244^T^)	1	Acidic mine drainage	Kishimoto et al., 1991	–	100	✓
*Occallatibacter savannae* A2-1c^T^ (= DSM 25170^T^)	1	Savannah soil	Foesel et al., 2016	–	100	–
*Occallatibacter riparius* 277^T^(= DSM 25168^T^)	1	River bank soil	Foesel et al., 2016	–	100	–
*Occallatibacter riparius* 307 (= DSM 25169)	1	River bank soil	Foesel et al., 2016	–	100	–
*Acidobacteriaceae* bacterium A2-4c	1	Savannah soil	Sinninghe Damsté et al., 2011	1–13	87–99	✓
*Acidicapsa borealis* KA1^T^ (= DSM 23886^T^)	1	*Spaghnum* peat	Kulichevskaya et al., 2012	–	100	✓
*Acidicapsa ligni* WH120^T^ (= LMG 26244^T^)	1	Decaying wood	Kulichevskaya et al., 2012	–	100	✓
*Acidicapsa* sp. CE1	1	*Spaghnum* peat	Pankratov, 2012	n.a.	n.a.	✓
*Ca*. Koribacter versatilis Ellin345 (= DSM 22529)	1	Pasture soil	Joseph et al., 2003	4–48	52–96	✓
*Terriglobus roseus* KBS 63^T^(= DSM 18391^T^)	1	Agricultural soil	Eichorst et al., 2007	1	99	✓
*Terriglobus* sp. KMR (= ATCC BAA-1395)	1	*Spaghnum* peat	Pankratov et al., 2008	n.a.	n.a.	–
*Granulicella pectinivorans* TPB6011^T^ (= DSM 21001^T^)	1	*Spaghnum* peat	Pankratov and Dedysh, 2010	n.a.	n.a.	✓
*Granulicella aggregans* TPB6028^T^ (= DSM 25274^T^)	1	*Spaghnum* peat	Pankratov and Dedysh, 2010	2	98	✓
*Granulicella paludicola* LCBR	1	*Cladonia sp*.	Pankratov and Dedysh, 2010	n.a.	n.a.	✓
*Granulicella paludicola* OB1010^T^(= DSM 22464^T^)	1	*Spaghnum* peat	Pankratov and Dedysh, 2010	n.a.	n.a.	✓
*Bryocella elongata* SN10^T^ (= DSM 22489^T^)	1	*Spaghnum* peat	Dedysh et al., 2012	n.a.	n.a.	✓
*Telmatobacter bradus* TPB6017^*T*^ (= DSM 23630^T^)	1	*Spaghnum* peat	Pankratov et al., 2012	n.a.	n.a.	–
*Telmatobacter* sp. 15–8A	1		Dunfield, Unpublished	n.a.	n.a.	✓
*Telmatobacter* sp. 15–28	1		Dunfield, Unpublished	n.a.	n.a.	✓
*Paludibaculum fermentans* P105^T^ (= DSM 26340^T^)	3	Littoral wetland	Kulichevskaya et al., 2014	3	97	✓
*Ca*. Solibacter usitatus Ellin6076	3	Pasture soil	Joseph et al., 2003	2	98	✓
*Bryobacter aggregatus* MPL3^T^ (= DSM 18758^T^)	3	*Spaghnum* peat	Kulichevskaya et al., 2010	–	100	✓
*Bryobacter aggregatus* MPL1011	3	*Spaghnum* peat	Kulichevskaya et al., 2010	n.a.	n.a.	✓
*Chloracidobacterium thermophilum* B^T^ (= ATCC BAA-2647)	4	Hot spring	Tank and Bryant, 2015	–	100	–
*Pyrinomonas methylalipathogenes* K22^T^ (= DSM 25857^T^)	4	Geothermal soil	Crowe et al., 2014	–	–	–
*Blastocatella fastidiosa* A2-16^T^ (= DSM 25172^T^)	4	Pastureland soil	Foesel et al., 2013	–	–	–
*Brevitalea aridisoli* Ac_11_E3^T^ (= DSM 27934^T^)	4	Savannah soil	Wüst et al., 2016	–	–	–
*Stenotrophobacter terrae* Ac_28_D10^T^ (= DSM 26560^T^)	4	Savannah soil	Pascual et al., 2015	n.a.	n.a.	–
*Aridibacter kavangonensis* Ac_23_E3^T^ (= DSM 26558^T^)	4	Fallow soil	Huber et al., 2014	–	–	–
*Aridibacter famidurans* A22_HD_4H^T^ (= DSM 26555^T^)	4	Savannah soil	Huber et al., 2014	n.a.	n.a.	–
*Vicinamibacter silvestris* Ac_5_C6^T^ (= DSM 29464^T^)	6	Savannah soil	Huber et al., 2016	–	–	–
*Luteitalea pratensis* HEG_-6_39 (= DSM 100886^T^)	6	Grassland soil	Vieira et al., 2017	–	–	–
*Holophaga foetida* TMBS4^T^ (= DSM 6591^T^)	8	Anoxic mud	Liesack et al., 1994	–	–	–
*Geothrix fermentans* H-5^T^ (= DSM 14018^T^)	8	Aquifier	Coates et al., 1999	–	–	–
*Thermotomaculum hydrothermale* AC55 (= DSM 24660)	10	Hydrothermal vent	Izumi et al., 2012	–	–	–
*Thermoanaerobaculum aquaticum* MP01^T^ (= DSM 24856^T^)	23	Hot spring	Losey et al., 2013	n.a.	n.a.	–

–*, Absent; n.a. = not analyzed, ✓, present*.

### Lipid analysis

For the detection of the presence of biohopanoids, lyophilized cells were directly treated with periodic acid/sodium borohydride to convert polyfunctionalized biohopanoids into GC-amenable hopanoid alcohols following procedure 2 described by Rohmer et al. (1984) with some modifications. Lyophilized cells (ca. 10 mg) were stirred with 1 ml of a solution of periodic acid (30 mg) in tetrahydrofuran/water (8:1, v/v) at room temperature for 1 h. After addition of water (1 ml), the lipids were extracted three times with dichloromethane (DCM; 2 ml), and the solution was dried over anhydrous Na_2_SO_4_, and evaporated to dryness. The residue was treated with 20 mg of NaBH_4_ (100 mg), in 1 ml methanol by stirring at room temperature for 1 h. After addition of a solution of KH_2_PO_4_ (1 ml, 200 mM), the hopanols were extracted with DCM. The obtained reaction mixture was methylated with diazomethane and separated over a small column with activated Al_2_O_3_ into an apolar and a polar fraction using DCM and DCM-MeOH (2:1, v/v) as eluent, respectively. The polar fractions were silylated with N,O-bis(trimethylsilyl)-fluoroacetamide in pyridine at 60°C for 20 min and analyzed by GC and GC-MS. The distribution of hopanols was obtained by integration of the appropriate peaks.

The lyophilized cells of strain A2-4c were also extracted ultrasonically with DCM/MeOH (2:1, v/v) (× 3) and the extract was subsequently subjected to the Rohmer degradation to compare the yield of the generation of hopanols from the extract as compared to direct treatment of the cell material. To this end, an internal standard (6,6-d_2_-3-methylheneicosane) was added to quantify the hopanols for the two different ways of treatment.

GC was performed using a Hewlett–Packard 6890 instrument, equipped with an on-column injector. A fused silica capillary column (25 m × 0.32 mm) coated with CP-Sil 5 (film thickness 0.12 μm) was used with helium as carrier gas. Samples were dissolved in ethyl acetate and injected at 70°C and the oven was programmed to 130°C at 20°C/min and then at 4°C/min to 320°C at which it was held for 10 min. GC-MS analyses were performed on a Finnigan Trace DSQ mass spectrometer operated at 70 eV with a mass range of m/z 40–800 and a cycle time of 1.7 s. The gas chromatograph was equipped with a fused silica capillary column as described above for GC. The carrier gas was helium and the same oven temperature program as for GC was used.

### Identification and phylogeny of genes of the BHP biosynthetic pathway

Biosynthetic genes involved in the BHP pathway were identified in acidobacterial (meta)genomes with PSI-BLAST (Position-Specific iterated BLAST) searches at the protein level (www.ncbi.com) using two iteration steps using in most cases the annotated enzymes from *Acidobacterium capsulatum* as query sequences. These *A. capsulatum* query sequences were first identified by PSI-BLAST searches of BHP biosynthesis genes annotated in *R. palustris, Zymomonas mobilis, Burkholderia cenocopacia, M. capsulatus*, and *Methylobacterium extorquens*, species where BHP biosynthetic genes have been assigned (e.g., Perzl et al., 1998; Bradley et al., 2010; Welander et al., 2010, 2012; Neubauer et al., 2015; Schmerk et al., 2015).

Nearly complete 16S rRNA gene sequences of the acidobacterial strains and (meta)genomes obtained from environmental samples discussed in the text, were obtained from the ARB SILVA database (https://www.arb-silva.de/) and from the (meta)genomes listed in Table 3, and aligned with ClustalW (Thompson et al., 1994). A phylogenetic tree was generated with MEGA 6 (Tamura et al., 2013) using the Neighbor-joining method (Saitou and Nei, 1987); bootstrapping values were based on 1,000 repetitions and are shown next to the branches (Felsenstein, 1985). The evolutionary distances were computed using the Jukes-Cantor method (Jukes and Cantor, 1969). The analysis involved 72 nucleotide sequences, and a total of 1593 positions in the final dataset. Putative and annotated partial homologs of Shc, HpnC, hpnD, and FdpT proteins were aligned by Muscle (Edgar, 2004) in Mega6 software (Tamura et al., 2013) and edited manually. Phylogenetic reconstruction was performed by maximum likelihood in PhyML v3.0 (Guindon et al., 2010) using the best model according to AIC indicated by ProtTest 2.4 (Abascal et al., 2005) as indicated in the figure legends.

## Results

**Figure 1:**
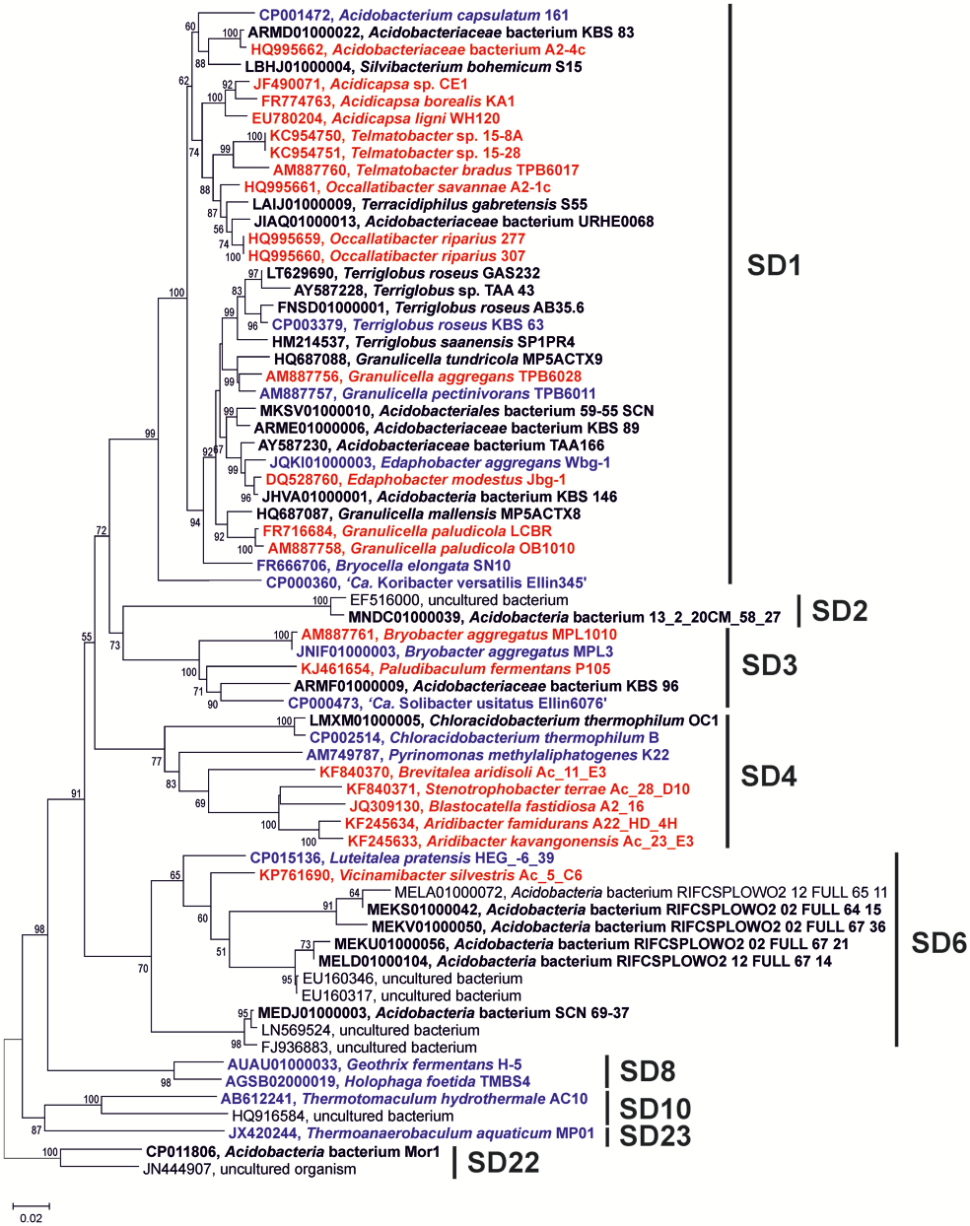
Phylogenetic tree of the nearly complete 16S rRNA gene sequences of the *Acidobacteria* discussed in the text. The percentage of replicate trees in which the associated taxa clustered together in the bootstrap test (1,000 replicates) are shown next to the branches. Scale bar indicates 2% sequence divergence. Names in red bold indicate strains in which presence of BHP has been assessed in cultures; names in black bold indicate strains in which the genome has been screened for the presence of the genes discussed in the text, names in blue bold indicate strains in which both the BHP and genome screening has been performed.

Thirty-eight *Acidobacteria* strains distributed over seven subdivisions (SDs 1, 3, 4, 6, 8, 10, and 23) were analyzed for the presence of C_30_ hopenes and BHPs, and specifically, for the presence of methylated hopanoids. Figure 1 depicts their phylogenetic relationship based on their 16S rRNA gene.

### Detection of BHPs in acidobacterial cultures

**Figure 2:**
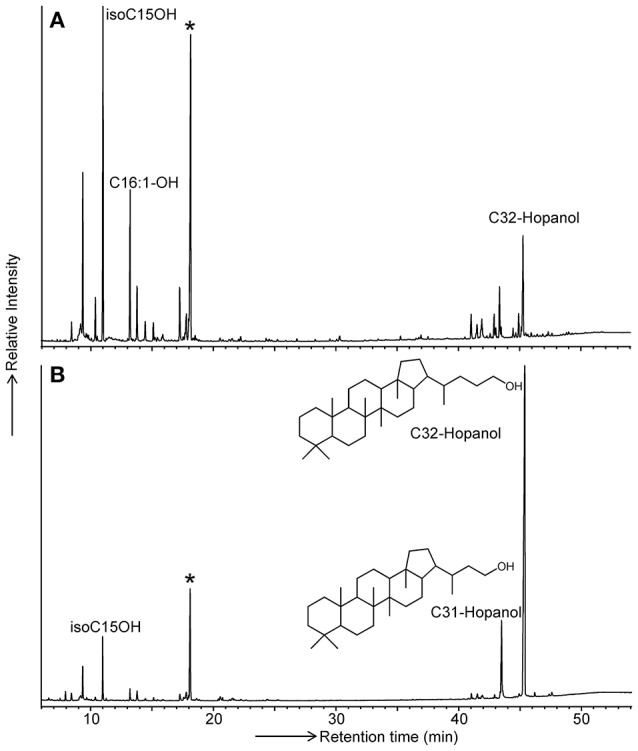
GC traces of the products formed by the Rohmer treatment of **(A)** the total extract and **(B)** total cell material of *Acidobacteriaceae* bacterium A2-4c. Released hopanoids are present in the form of hopanols (analyzed as their TMS derivatives). Bacteriohopanetetrol derivatives are transformed by the Rohmer treatment into the C_32_ hopanol, bacteriohopanepentol derivatives are transformed into the C_31_ hopanol. The star indicates the internal standard added prior to analysis in a fixed concentration. Since the peak areas of the hopanols relative to the internal standard in **(B)** is much larger, the hopanol yield is much higher when the Rohmer reaction is applied to total biomass.

It has previously been demonstrated that specific membrane lipids of *Acidobacteria* (i.e., *iso* diabolic acid and 13,16-dimethyl-28-glyceryloxy-octacosanoic acid) can only be released by direct acid hydrolysis of lyophilized cells and not directly by a modified Bligh-Dyer extraction (Sinninghe Damsté et al., 2011, 2014). This was interpreted to indicate that these membrane-spanning lipids that comprised a substantial fraction of the membrane lipids contained large and potentially very polar head groups, making them inaccessible for solvent extraction. Since BHPs often reside in the membrane, where they act as rigidifiers (e.g., Rezanka et al., 2010), we suspected that the classical way of BHP analysis by extraction might miss a substantial fraction of BHPs in *Acidobacteria*. To test this hypothesis, we modified a commonly applied technique for the analysis of BHP derivatives, i.e., specific oxidation with periodic acid followed by reduction with sodium borohydride to convert complex polyfunctionalized BHPs into easy-to-analyze hopanoid alcohols (Rohmer et al., 1984) and applied this technique to lyophilized cells instead of to the extract of the cells (see experimental). Comparison of the yield of extended (i.e., C_31_ and C_32_) hopanols from *Acidobacteriaceae* bacterium A2-4c, belonging to SD1, revealed that direct treatment on lyophilized cells resulted in a substantial increase in yield (i.e., by approximately one order of magnitude; Figure 2). This indicates that, in addition to *iso* diabolic acid and its derivatives, also BHP derivatives are difficult to extract from acidobacterial cells. Consequently, we analyzed our strains by application of the “Rohmer method” on lyophilized cells.

### Extended hopanols formed by rohmer degradation of intact cells

Cell material of 24 different strains of six subdivisions (SDs 1, 3, 4, 6, 8, and 10) of the *Acidobacteria* were subjected to the Rohmer reaction to test the presence of BHPs. C_32_, and in some cases C_31_ hopanols, were detected in all strains of SD 1 and 3 but not in SD 4, 6, 8, and 10 (Table 1). An exception was the photosynthetic thermophilic acidobacterium *C. thermophilum* B, where bishomohopanol was detected, in agreement with the reported occurrence of BHT derivatives in this strain (Costas et al., 2012b). In almost all strains only bishomohopanol was detected; this hopanol is derived from the oxidation of tetrafunctionalized BHPs and subsequent reduction (Rohmer et al., 1984). Five strains (i.e., *Acidobacteriaceae* bacterium A2-4c, “*Ca*. Koribacter versatilis Ellin345,” *Terriglobus roseus* KBS63^T^, *Paludibaculum fermentans* P105^T^, and “*Ca*. Solibacter usitatus Ellin6076;” Table 1) contained variable relative abundances (up to 45%) of homohopanol, derived from pentafunctionalized BHPs.

**Figure 3:**
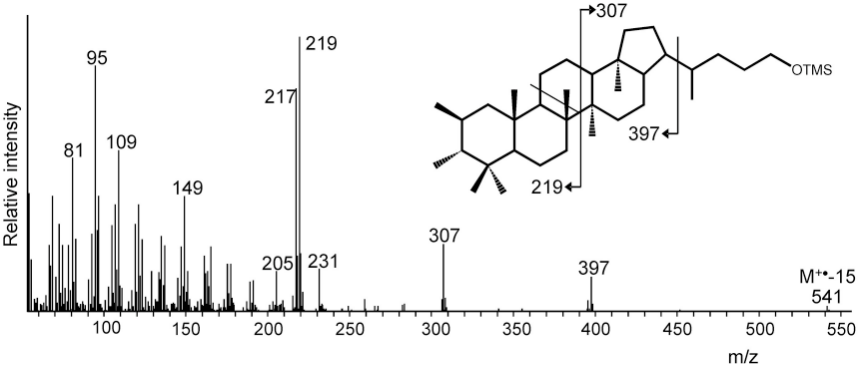
Mass spectrum of a putative dimethylated bishomohopanol (as TMS derivative) formed by Rohmer degradation of total cell material of “*Ca*. Koribacter versatilis” Ellin345. The methylation at position C-2 and C-3 is unprecedented but supported by the indicated mass spectral fragmentation and the presence of both the *hpnP* and *hpnR* genes in the genome of “*Ca*. K. versatilis Ellin345” (Table 2). The indicated stereochemistry of the additional methyl groups is hypothetical.

Methylated extended hopanols (derived from 2-methyl or 3-methyl BHPs) were only detected in one (out of three) batch cultures of “*Ca*. Koribacter versatilis Ellin345.” In this case, an extended hopanol was tentatively identified as 2,3-dimethyl-bishomohopanol on the basis of its mass spectral features (Figure 3) and relative retention time. It comprised ca. 9% of the hopanols released by the Rohmer degradation.

### C_30_ hopanoids in *Acidobacteria*

All 24 strains analyzed for the presence of BHPs and 14 others were also examined for the presence of the C_30_ hopanoids. The occurrence of hop-17(21)-ene is reported in Table 1. This hopanoid is formed from both diploptene [hop-27(29)-ene] and diplopterol upon acid hydrolysis, the extraction method that was typically applied (Sinninghe Damsté et al., 2011, 2014). If cells were extracted with a modified Bligh-Dyer protocol, these two latter hopanoids (mostly diploptene) were detected instead of hop-17(21)-ene. In general, strains that do produce BHPs also contain C_30_ hopanoids although there are some exceptions (Table 1). C_30_ hopanoids were not detected in strains that do not produce BHPs (Table 1). The C_30_ hopanoid data reveal that hopanoid synthesis occurs in all strains of the SD1 *Acidobacteria* analyzed.

**Table 2:** Presence of genes involved in the biosynthesis of hopanoids in genomes of cultivated *Acidobacteria* based on BLAST searches of the protein sequence.

***Acidobacterial* genome^a^**	**Size (Mb)**	**SD**	**Detected genes^b^**																							
				**Non-mevalonate pathway**								**Squalene synthesis**					**BHP synthesis**						**Meth**.		**Put**.	
			***aceE***	***dxs***	***dxr***	***ispD***	***ispE***	***ispF***	***ispG***	***ispH***	***ispA***	***hpnD***	***hpnC***	***hpnE***	***fdpT***	***shc***	***hpnH***	***hpnG***	***hpnI***	***hpnK***	***hpnJ***	***hpnO***	***hpnP***	***hpnR***	***hpnA***	***hpnB***
***Acidobacterium capsulatum*** 161	4.1	1	✓	✓	✓	✓	✓	✓	✓	✓	✓	✓	✓	✓	—	✓	✓	✓	✓	—	✓	✓	—	—	✓	—
*Silvibacterium bohemicum* S15	6.5	1	✓	✓	✓	✓	✓	✓	✓	✓	✓	✓	—	—	✓	✓	✓	✓	✓	—	✓	✓	—	—	✓	—
*Acidobacterium ailaaui* PMMR2	3.7	1	✓	✓	✓	✓	✓	✓	✓	✓	✓	✓	✓	✓	—	✓	—	✓	✓	—	✓	✓	—	—	✓	—
*Acidobacteriaceae* bacterium KBS 83	6.3	1	✓	✓	✓	✓	✓	✓	✓	✓	✓	✓	✓	✓	—	✓	✓	✓	✓	—	✓	✓	—	—	✓	—
*Acidobacteriaceae* bacterium URHE0068	6.7	1	—	✓	✓	✓	✓	✓	✓	✓	✓	✓	✓	✓	—	✓	✓	✓	✓	—	✓	✓	—	—	✓	✓
*Terracidiphilus gabretensis* S55	5.3	1	—	✓	✓	✓	✓	✓	✓	✓	✓	✓	✓	✓	—	✓	✓	✓	✓	—	✓	✓	—	—	✓	—
*Acidobacteriaceae* bacterium KBS 89	6.0	1	✓	✓	✓	✓	✓	✓	✓	✓	✓	✓	✓	—	✓	✓	✓	✓	✓	—	✓	✓	—	—	✓	—
*Granulicella mallensis* MP5ACTX8	6.2	1	✓	✓	✓	✓	✓	✓	✓	✓	✓	✓	—	—	✓	✓	✓	✓	✓	—	✓	✓	—	—	✓	✓
*Acidobacteria* bacterium KBS 146	5.0	1	✓	✓	✓	✓	✓	✓	✓	✓	✓	✓	✓	✓	—	✓	✓	✓	✓	—	✓	✓	—	—	✓	—
***Edaphobacter aggregans*** Wbg-1	8.2	1	✓	✓	✓	✓	✓	✓	✓	✓	✓	✓	✓	✓	2	✓	✓	✓	✓	—	✓	✓	—	—	✓	✓
*Terriglobus saanensis* SP1PR4	5.1	1	✓	✓	✓	✓	✓	✓	✓	✓	✓	✓	✓	✓	—	✓	✓	✓	✓	—	✓	—	—	—	✓	—
*Terriglobus roseus* AB35.6	4.8	1	✓	✓	✓	✓	✓	✓	✓	✓	✓	✓	✓	✓	—	✓	✓	✓	✓	—	✓	—	—	—	✓	—
***Terriglobus roseus*** DSM 18391	5.2	1	✓	✓	✓	✓	✓	✓	✓	✓	✓	✓	✓	✓	—	✓	✓	✓	✓	—	✓	—	—	—	✓	—
*Terriglobus sp*. TAA 43	4.9	1	✓	✓	✓	✓	✓	✓	✓	✓	✓	✓	✓	✓	—	✓	✓	✓	✓	—	✓	—	—	—	✓	✓
*Terriglobus roseus* GAS232	4.8	1	✓	✓	✓	✓	✓	✓	✓	✓	—	✓	✓	✓	—	✓	✓	✓	✓	—	✓	—	—	—	✓	✓
***Granulicella pectinivorans*** TPB6011	5.3	1	✓	✓	✓	✓	✓	✓	✓	✓	✓	✓	✓	✓	—	2	✓	✓	✓	—	✓	—	—	—	✓	—
*Acidobacteriaceae* bacterium TAA166	6.1	1	✓	✓	✓	✓	✓	✓	✓	✓	✓	✓	—	—	✓	✓	✓	✓	✓	—	✓	—	—	—	✓	—
*Granulicella tundricola* MP5ACTX9	5.5	1	✓	✓	✓	✓	✓	✓	✓	✓	✓	✓	✓	✓	—	✓	✓	✓	✓	—	✓	—	—	—	✓	—
***Bryocella elongata*** SN10	n.d.	1	✓	✓	✓	✓	✓	✓	✓	✓	✓	✓	✓	✓	—	✓	✓	✓	✓	—	✓	—	—	—	✓	—
***Ca***. **Koribacter versatilis** Ellin345	5.6	1	✓	—	✓	✓	✓	✓	✓	✓	✓	✓	✓	✓	—	✓	✓	✓	✓	—	✓	✓	✓	✓	✓	—
*Acidobacteriaceae* bacterium KBS96	6.7	3	✓	—	✓	✓	✓	✓	✓	✓	✓	✓	✓	✓	—	✓	✓	✓	✓	—	✓	✓	—	—	✓	✓
**Ca. Solibacter usitatus** Ellin6076	10.0	3	✓	✓	✓	✓	✓	✓	✓	✓	✓	✓	✓	✓	—	2	✓	✓	✓	—	✓	✓	—	—	✓	✓
***Bryobacter aggregatus*** MPL3	5.7	3	✓	—	✓	✓	✓	✓	✓	✓	✓	✓	—	✓	—	✓	✓	✓	✓	—	✓	✓	—	—	✓	—
***Chloracidobacterium thermophilum*** B	3.6	4	✓	—	✓	✓	✓	✓	✓	✓	✓	✓	✓	✓	✓	✓	✓	✓	✓	✓	✓	✓	—	—	✓	—
*Chloracidobacterium thermophilum* OC1	3.6	4	✓	—	✓	✓	✓	✓	✓	✓	✓	✓	✓	✓	✓	✓	✓	✓	✓	✓	✓	—	—	—	✓	—
***Pyrinomonas methylaliphatogenes*** K22	3.8	4	—	✓	✓	✓	—	✓	✓	✓	✓	—	—	—	—	—	—	—	—	—	—	✓	—	—	—	—
***Luteitalea pratensis*** HEG_-6_39	7.5	6	✓	—	✓	✓	✓	✓	✓	✓	✓	✓	✓	✓	—	—	—	—	—	—	—	✓	—	—	—	—
***Holophaga foetida*** TMBS4	4.1	8	—	✓	✓	✓	✓	✓	✓	✓	✓	—	—	—	—	—	—	—	—	—	—	—	—	—	—	—
***Geothrix fermentans*** H-5	3.3	8	—	✓	✓	✓	✓	✓	✓	✓	✓	—	—	—	—	—	—	—	—	—	—	—	—	—	—	—
***Thermotomaculum hydrothermale*** AC55	n.d.	10	—	✓	✓	✓	✓	✓	✓	✓	✓	—	—	—	—	—	—	—	—	—	—	—	—	—	—	—
***Thermoanaerobaculum aquaticum*** MP-01	2.7	23	✓	—	✓	✓	✓	✓	✓	✓	✓	—	—	—	—	—	—	—	—	—	—	—	—	—	—	—

*✓, Present; 2, two copies detected, —, absent, Meth., methylation; Put., putative*.

a *For species in bold BHP analysis was performed (see Table 1)*.

b *Genbank accession see Tables S2–S6*.

### BHP biosynthetic genes in genomes of cultured *Acidobacteria*

**Figure 4:**
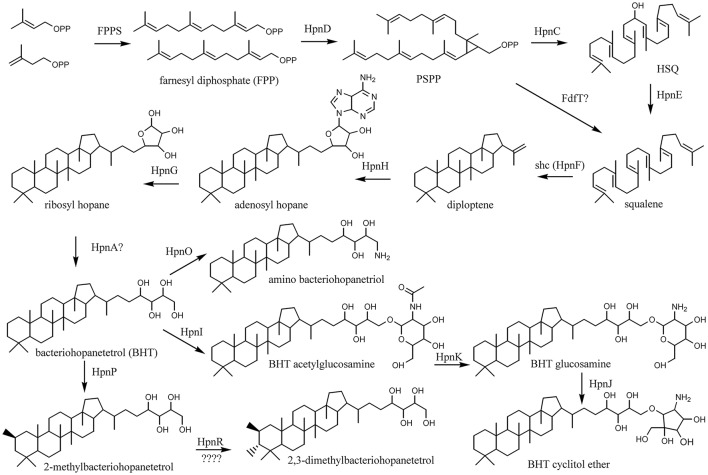
Biosynthetic scheme of synthesis of BHT derivatives.

To be able to compare the *in situ* production of BHPs with the biosynthetic potential at the genetic level, we screened available complete genomes of cultivated acidobacteria for biosynthetic genes involved in BHP production (see Figure 4 for the biosynthetic scheme). Thirty-one genomes from SDs 1, 3, 4, 6, 8, 10, and 23 were examined using protein BLAST searches. The results are listed in Table 2. In all acidobacteria investigated (almost) all genes encoding the MEP pathway of isoprenoid biosynthesis (*dxs, dxr, ispD, ispE, ispF, ispG, ispH*; Zhao et al., 2013) were detected. An exception is the *dxs* gene that was lacking in six species (Table 2). In *Escherichia coli* it has been demonstrated that a point mutation of the gene encoding the E1 subunit of the pyruvate dehydrogenase complex (*aceE*) can overcome the lack of *dxs* (Sauret-Güeto et al., 2006). In all of the acidobacterial species lacking *dxs, aceE* was detected instead. All examined acidobacterial genomes also encoded *ispA*, the gene encoding farnesyl diphosphate synthase. Therefore, all of the examined acidobacterial genomes have the genetic capacity to produce the isoprenoid C_15_ building block used in various biosynthetic pathways, including BHP synthesis (Figure 4).

**Figure 5:**
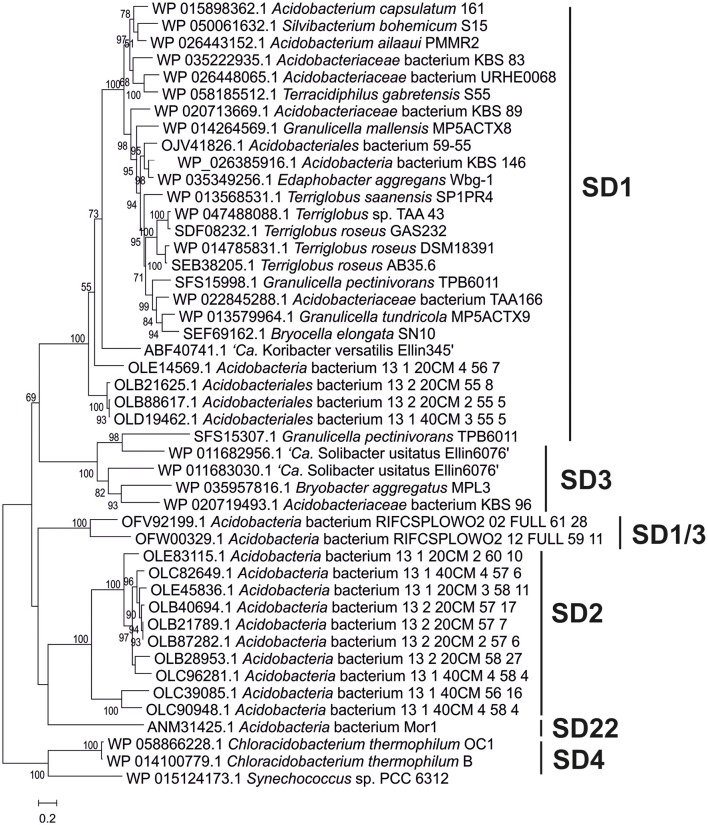
Phylogenetic tree of the Shc proteins in the acidobacterial genomes of cultures and environmental genomes. This tree was constructed using the maximum likelihood method with a LG model plus gamma distribution and invariant sites (LG+G+I). The analysis included 750 positions in the final dataset. The scale bar represents number of amino acid substitutions per site. Branch support was calculated with the approximate likelihood ratio test (aLRT) and values ≥50% are indicated on the branches. In general, it reveals the phylogeny that is also apparent from the 16S rRNA gene tree (Figure 1) with distinct clusters for the SD1, SD2, SD3, and SD22 clusters. The only Shc proteins encountered in *Acidobacteria* SD4 (i.e., in *C. thermophilum* B and OC1) differ substantially from those of other *Acidobacteria* and are more closely related to Shc proteins encountered in *Cyanobacteria* (e.g., *Synechoccus* sp.). The second Shc protein of the SD1 acidobacterium *G. pectinivorans* DSM21001 falls in SD3 and is most closely related to the second copy of Shc of “*Ca*. S. usitatus Ellin 6076.”

**Figure 6:**
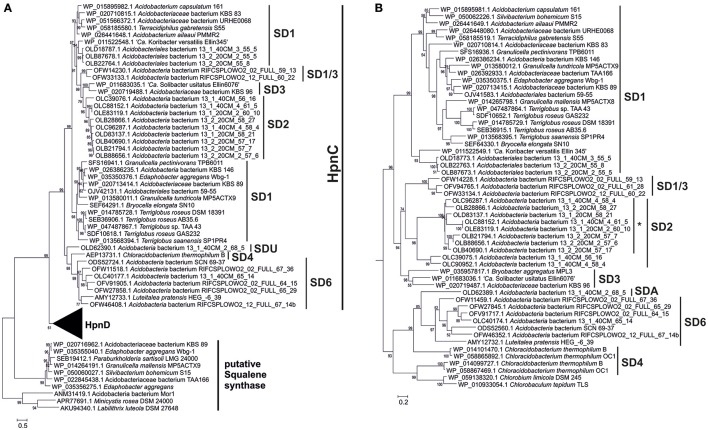
Phylogenetic tree of the HpnC, HpnD, and FdfT proteins in the acidobacterial genomes of cultures and environmental genomes. The tree was constructed using the maximum likelihood method with a LG model plus gamma distribution and invariant sites (LG+G+I). The analysis included 509 positions in the final dataset. The scale bar represents the number of amino acid substitutions per site. Branch support was calculated with the approximate likelihood ratio test (aLRT) and values ≥50% are indicated on the branches. The first part **(A)** of the tree shows the phylogeny of the HpnC and FdfT proteins; zoom in **(B)** showing the phylogeny of the HpnD proteins. The HpnC tree generally reveals the phylogeny that is also apparent from the 16S rRNA gene tree (Figure 1) with distinct clusters for the SD1, SD2, SD3, SD4, SD6, and SD1/3 acidobacteria. Five species of SD1 acidobacteria also contained an FdtT protein, which is only remotely related to the HpnC and HpnD proteins. These FdtT proteins are closely related to the FdtT proteins of *Betaproteobacteria* (e.g., *Parabulkholderia sartisoli*). The HpnC tree generally also reveals the phylogeny that is also apparent from the 16S rRNA gene tree (Figure 1) with distinct clusters for the SD1, SD2, SD3, and SD1/3 acidobacteria. The sequences in the SD2 cluster annotated with an asterisk are annotated as HpnC but in fact represent a fused protein HpnCD (see text and Figure 7). HpnC was considered until the amino acid position 279–339 up to the amino acids RAG/RTG/RVG, while the rest of the protein was cropped and used as a new entry in the alignment and phylogenetic tree. As observed in the Shc protein tree (this figure) the HpnD protein of the SD4 acidobacterium *C. thermophilum* is only distantly related to the HpnD protein of other acidobacteria. The closest relatives are HpnD proteins of green sulfur bacteria (e.g., *Chlorobium limicola* and *Chlorobaculum tepidum*).

The next step in the production of BHPs is the coupling of two molecules of farnesyl diphosphate and production of squalene. Recent evidence has revealed that in most bacteria this proceeds via a three-step mechanism (Figure 4) catalyzed by three different enzymes encoded by *hpnD, hpnC*, and *hpnE* (Pan et al., 2015). None of these genes were detected in *Acidobacteria* of SD8, SD10, and SD23 neither in *Pyrinomonas methylaliphatogenes* K22^T^ belonging to SD4 (Table 2), whereas all three genes were generally detected in the genomes of SD1 and SD3 species and in *C. thermophilum* B and OC1 (SD4) and in *Luteitalea pratensis* HEG_-6_39 (SD6) (Table 2). The genomes of all SD1 and SD3 species and *C. thermophilum* B and OC1 contained *shc*, encoding the enzyme responsible for the cyclization of squalene to form the hopanoid building block diploptene (Table 2; Figure 4). In *C. thermophilum* B and OC1 the detected *shc* genes are only remotely related to those of other *Acidobacteria* and much more closely affiliated with *shc* genes of *Cyanobacteria* (Figure 5). Two species (*G. pectinivorans* DSM 21001 and “*Ca*. S. usitatus Ellin 6076”) contained two copies of *shc* in their genome. In the *shc* phylogeny (Figure 5) both copies fall in the SD3 cluster. No *shc* gene could not be annotated in the remaining species (SDs 6, 8, 10, 23, and *P. methylaliphatogenes* K22; Table 2). In four SD1 genomes (i.e., those of *Silvibacterium bohemicum* S15, *Acidobacteriaceae bacterium* KBS 89, *Granulicella mallensis* MP5ACTX8^T^, and *Acidobacteriaceae* bacterium TAA166) in which *shc* could be annotated, not all of the three genes involved in squalene synthesis (*hpnD, hpnC*, and *hpnE*) were present (Table 2). However, in those genomes, we detected a gene annotated as a farnesyl diphosphate farnesyl transferase, which may be coding an alternative enzyme involved in squalene synthesis. This protein is highly homologous to proteins encoded by genes also annotated as farnesyl diphosphate farnesyl transferases present in the genomes of *Parabulkholderia* species, belonging to the *Betaproteobacteria* (Figure 6). In the two strains of *C. thermophilum* an additional gene copy of *hpnD* was also identified; it is phylogenetically most closely related to species of green sulfur bacteria (Figure 6).

All but one of the species harboring the *shc* gene also encode the two known genes involved in the conversion of diploptene to BHT (i.e., *hpnH* and *hpnG*). We suspect that *hpnA*, a sugar epimerase, is also involved in this conversion but there is no published experimental evidence for that (Table S1). The *hpnB* gene, for which experimental evidence is also lacking that it is involved in the BHP biosynthetic pathway, is present in only a limited number of the examined genomes, suggesting that it is not essential for BHP synthesis. This is in contrast to the *hpnA* gene, which is present in all acidobacteria possessing the genes required for BHT synthesis. In most SD1 *Acidobacteria hpnA* is also organized in a specific gene cluster together with *hpnG* (see below), which provides complementary circumstantial evidence for its potential role in BHP synthesis.

Only the genome of *C. thermophilum* B encodes all the genes (Table 2) required for the conversion of BHT into the composite BHP, BHT cyclitol ether (Figure 4). Other genomes contain some but not all of these genes (Table 2). The gene that is responsible for the last step of the conversion of BHT into amino bacteriohopanetriol (Figure 4), occurs in many of the examined strains but not all (Table 2). This gene was also detected in some acidobacteria that do not possess the *shc* gene. It is likely that this is caused by their close relatedness with other aminotransferases (e.g., *argD*; Welander et al., 2012).

Of special interest were the genes encoding enzymes responsible for methylation of the A-ring of BHPs. Welander et al. (2010) and Welander and Summons (2012) previously reported the presence of *hpnP* and *hpnR* in the genome of the acidobacterium “*Ca*. K. versatilis Ellin345,” which was confirmed by our BLAST searches (Table 2; *hpnP* WP_011523124.1 and *hpnR* WP_011523840.1; both with sequence identity of ca. 60% with the genes of *R. palustris* TIE-1 (Welander et al., 2010) and *M. capsulatus* (Welander and Summons, 2012), respectively). However, these genes were absent in all of the other examined acidobacterial genomes (Table 2).

**Figure 7:**
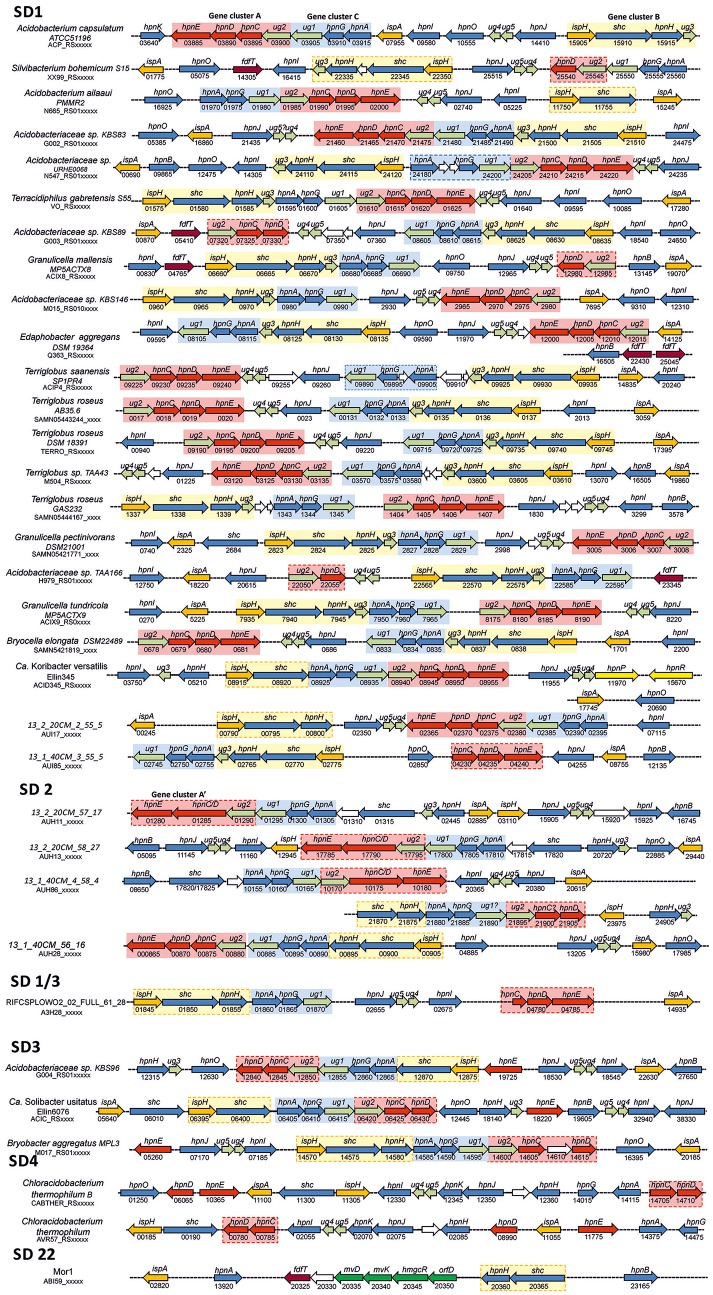
Location of BHP biosynthetic genes and gene clusters in the genomes of SD1, SD3, and SD4 *Acidobacteria* and a number of selected environmental genomes as determined by BLAST protein searches. Species were ordered as indicated by the phylogeny of the *shc* gene. Stippled lines indicate a distance between the genes. The numbers refer, in combination with the code below the species name, to the locus tag in the annotated genome from the NCBI database. These numbers typically increase by 5 for every next gene. Color codes of genes are: orange, genes involved in the biosynthesis of farnesyl diphosphate; red and dark red (putative), genes involved in the biosynthesis of squalene; blue, genes involved in BHP biosynthesis; yellow, genes involved in the methylation of BHPs; dark green, genes involved in the mevalonate pathway of isoprenoid biosynthesis, light green, genes often of unknown function associated with identified gene clusters. Names of genes refer to Table S1. Three gene clusters were often encountered and these are indicated in different background colors (red, blue, and yellow). When these cluster are indicated with a stippled box, these clusters have been slightly modified by the insertion or deletion of one or a few genes. ^*^There is only a nucleotide sequence corresponding to the open reading frame of *hpnC* of *C. thermophilum* B (WP_014101470.1) between amino acids 38–227.

The phylogeny of the detected BHP biosynthetic genes (e.g., Figures 5, 6) generally showed a similar clustering as that observed for the 16S rRNA gene phylogeny (Figure 1). The location of the BHP biosynthetic genes in the *Acidobacteria* genomes investigated showed some distinct gene clustering organization. Three clusters were identified (Figure 7). Cluster A is composed of *hpnC, hpnD*, and *hpnE*, preceded by a gene with an unknown function (*ug2*). Cluster B is composed of *ispA, shc*, and *hpnH*, followed by a gene with an unknown function (*ug3*). Lastly, cluster C is composed of *hpnA*, and *hpnG*, followed by another gene with an unknown function (*ug1*). In some cases these gene clusters have been expanded by the insertion of other genes or have lost genes (indicated by stippled lines in Figure 7). In *Terracidophilus gabretensis* S55^T^ these three clusters occur in one large concatenated cluster, including *hpnJ* (Figure 5). In other SD1 acidobacteria, this ordering is seen to an increasingly less extent. For example, in *Acidobacteriaceae* sp. KBS83 the ordering is almost the same as in *T. gabretensis* S55^T^ except that there is an insertion of two additional genes between clusters A-C and *hpnJ* (Figure 7). On the other side of the spectrum, there are SD1 acidobacteria that lack one or two of the gene clusters, either because they miss one or more of the genes of a gene cluster (e.g., *hpnC* and/or *hpnE* in cluster A) or because the cluster was split into two parts (e.g., gene cluster B in “*Ca*. Koribacter versatilis Ellin345”). In SD3 and the one species of SD4 that possesses genes for BHP synthesis (*C. thermophilum* B and OC1), the ordering of genes as observed for SD1 acidobacteria is much less apparent. Only gene cluster C could be recognized in SD3 acidobacteria (Figure 7). The localization of the other genes that occur predominantly clustered in SD1 was found to be more scattered.

### Identification of genes involved in BHP biosynthesis in acidobacterial genomes from the environment

Recently, a number of environmental genomes of acidobacteria obtained from environmental metagenomic datasets (e.g., soil, groundwater, wastewater sludge) has become publically available (e.g., Anantharaman et al., 2016; Butterfield et al., 2016; Speth et al., 2016). Some of these genomes are from acidobacteria that phylogenetically do not fall in phyla for which cultivated members are known and allow to search for the more widespread occurrence of BHP synthesis in *Acidobacteria*. Accordingly, we examined the presence of genes involved in BHP biosynthesis in 42 acidobacterial environmental genomes (Table 3). A complication with this analysis is that these environmental genomes are typically not complete and, thus, the absence of a gene should be taken with caution. The genomes are classified as indicated in Table 3 based on 16S rRNA gene when available (Figure 1) and on the phylogenetic clustering of the other genes studied (e.g., Figures 5, 6). Four of them fall in SD1 (with three of the most closely related to “*Ca*. K. versatilis Ellin345”), 11 in SD2, 3 in SD4, 10 in SD6; SD22, and SD23 are each represented by one genome. The other genomes can't clearly be assigned to a SD since they lack 16S rRNA gene (or contain only a small part of it) and fall in phylogenomic clusters with no cultured relatives. Five genomes cluster in a group that is phylogenetically (based on the genes of the BHP pathway) closely related to SD1 and SD3 (labeled 1/3 in Table 3). A group of two genomes belong to a cluster closely related to SD3 and SD4 (labeled 3/4), and there are four genomes that cannot be classified in this way and are labeled U in Table 3.

**Table 3:** Presence of genes involved in the biosynthesis of hopanoids in (partial) genomes of *Acidobacteria* from environmental metagenomes based on BLAST searches of the protein sequence.

**Environmental *Acidobacteria*l genome**	***SD***	**Detected genes^a^**																							
			**Non-mevalonate pathway**								**Squalene synthesis**					**BHP synthesis**						**Meth**.		**Put**.	
		***aceE***	***dxs***	***dxr***	***ispD***	***ispE***	***ispF***	***ispG***	***ispH***	***ispA***	***hpnD***	***hpnC***	***hpnE***	***fdpT***	***shc***	***hpnH***	***hpnG***	***hpnI***	***hpnK***	***hpnJ***	***hpnO***	***hpnP***	***hpnR***	***hpnA***	***hpnB***
59–55	1	—	✓	✓	✓	—	✓	✓	✓	✓	✓	✓	✓	—	✓	✓	—	✓	—	✓	—	—	—	✓	**—**
13_1_20CM_4_56_7	1	—	—	✓	**2**	✓	✓	✓	—	—	—	—	—	—	✓	✓	—	—	—	—	✓	—	—	—	**—**
13_1_40CM_3_55_5	1	—	—	✓	—	✓	—	—	—	✓	✓	✓	✓	—	✓	—	✓	—	—	✓	—	—	—	✓	✓
13_2_20CM_2_55_5	1	—	—	✓	✓	—	✓	✓	✓	✓	✓	✓	✓	—	✓	✓	✓	✓	—	✓	—	—	—	✓	**—**
13_2_20CM_55_8	1	—	✓	✓	✓	—	✓	✓	✓	✓	✓	✓	✓	—	✓	—	—	✓	—	—	—	—	—	—	**—**
13_1_20CM_2_60_10	2	—	✓	—	—	—	—	—	—	✓	✓	✓	✓	—	✓	—	✓	—	—	—	✓	—	—	✓	**—**
13_1_20CM_3_58_11	2	—	—	✓	✓	—	✓	✓	—	✓	—	—	—	—	✓	—	—	—	—	—	✓	—	—	—	✓
13_1_20CM_58_21	2	—	✓	—	✓	✓	—	—	✓	✓	✓	✓	✓	—	—	✓	✓	—	—	—	—	—	—	✓	✓
13_1_40CM_4_57_6	2	—	—	—	—	—	—	—	—	—	—	—	—	—	✓	—	✓	**2**	—	✓	—	—	—	✓	✓
13_1_40CM_4_58_4	2	—	✓	—	✓	✓	✓	—	✓	✓	**2**	✓	✓	—	**2**	**2**	✓	✓	—	✓	—	—	—	✓	✓
13_1_40CM_4_61_5	2	—	✓	✓	—	—	—	—	—	—	✓	✓	✓	—	—	✓	✓	—	—	—	—	—	—	✓	✓
13_2_20CM_2_57_6	2	✓	✓	✓	✓	✓	✓	✓	✓	—	—	✓	✓	—	✓	✓	✓	—	—	—	✓	—	—	✓	✓
13_2_20CM_57_17	2	✓	✓	✓	✓	✓	✓	✓	✓	✓	✓	✓	✓	—	✓	✓	✓	—	—	✓	—	—	—	✓	✓
13_2_20CM_57_7	2	—	✓	✓		✓	✓	✓		✓	✓	✓	—	—	✓	✓	✓	✓	—	✓	✓	—	—	✓	**—**
13_2_20CM_58_27	2	✓	✓	✓	✓	✓	✓	—	✓	✓	✓	✓	✓	—	✓	✓	✓	✓	—	✓	✓	—	—	✓	✓
13_1_20CM_56_16	2′	✓	✓	✓	✓	✓	✓	—	✓	✓	✓	✓	✓	—	✓	✓	✓	**3**	—	✓	✓	—	—	✓	**—**
RIFCSPLOWO2_02_FULL_59_13	1/3	✓	—	✓	✓	—	✓	✓	✓	✓	✓	✓	—	—	—	—	✓	✓	—	✓	✓	—	—	✓	**—**
RIFCSPLOWO2_02_FULL_61_28	1/3	✓	—	—	✓	✓	✓	—	✓	✓	✓	✓	✓	—	✓	✓	✓	**2**	—	✓	✓	—	—	✓	**—**
RIFCSPLOWO2_12_FULL_54_10	1/3	✓	—	✓	✓	✓	✓	—	✓	✓	—	—	✓	—	—	✓	—	—	—	✓	—	—	—	—	**—**
RIFCSPLOWO2_12_FULL_59_11	1/3	—	—	—	✓	—	✓	—	✓	✓	—	—	—	—	✓	✓	✓	—	—	—	✓	—	—	✓	**—**
RIFCSPLOWO2_12_FULL_60_22	1/3	✓	—	—	✓	—	✓	✓	—	✓	✓	✓	✓	—	—	—	✓	✓	—	—	✓	—	—	—	**—**
RIFCSPHIGHO2_02_FULL_67_57	3/4	—	—	✓	✓	—	—	—	—	✓	—	—	—	—	—	—	—	—	—	—	—	—	—	—	**—**
RIFCSPHIGHO2_12_FULL_67_30	3/4	—	—	—	✓	✓	✓	—	—	✓	—	—	—	—	—	—	—	—	—	—	—	—	—	—	**—**
OLB17	4	—	—	✓	—	✓	—	✓	✓	✓	—	—	—	—	—	—	—	—	—	—	✓	—	—	—	**—**
13_1_20CM_3_53_8	4	—	✓	✓	✓	✓	✓	✓	✓	✓	—	—	—	—	—	—	—	—	—	—	✓	—	—	—	**—**
RBG_16_70_10	4	—	—	—	✓	—	✓	✓	✓	✓	—	—	—	—	—	—	—	—	—	—	—	—	—	—	**—**
RIFCSPLOWO2_02_FULL_64_15	6	✓	—	✓	✓	✓	✓	✓	✓	✓	✓	✓	—	—	—	—	—	—	—	—	—	—	—	—	**—**
RIFCSPLOWO2_02_FULL_65_29	6	✓	—	✓	✓	✓	✓	✓	✓	✓	✓	✓	✓	—	—	—	—	—	—	—	✓	—	—	—	**—**
RIFCSPLOWO2_02_FULL_67_36	6	✓	—	✓	✓	✓	✓	✓	✓	✓	✓	✓	✓	—	—	—	—	—	—	—	✓	—	—	—	**—**
RIFCSPLOWO2_02_FULL_67_21	6	✓	—	✓	✓	—	✓	✓	✓	✓	—	—	—	—	—	—	—	—	—	—	✓	—	—	—	**—**
RIFCSPLOWO2_02_FULL_68_18	6	✓	—	✓	✓	—	✓	✓	✓	✓	—	—	—	—	—	—	—	—	—	—	—	—	—	—	**—**
RIFCSPLOWO2_12_FULL_67_14b	6	✓	—	—	✓	✓	✓	✓	✓	✓	✓	✓	✓	—	—	—	—	—	—	—	✓	—	—	—	**—**
13_1_20CM_2_65_9	6	—	—	✓	✓	—	✓	—	✓	✓	—	—	—	—	—	—	—	—	—	—	—	—	—	—	**—**
13_1_40CM_2_64_6	6	—	—	—	✓	—	✓	**2**	—	✓	—	—	—	—	—	—	—	—	—	—	—	—	—	**—**	**—**
13_1_40CM_65_14	6	✓	✓	—	✓	✓	✓	—	✓	✓	✓	—	✓	—	—	—	—	—	—	—	—	—	—	—	✓
SCN 69—37	6	✓	—	✓	✓	✓	✓	✓	✓	✓	✓	✓	✓	—	—	—	—	—	—	—	—	—	—	—	**—**
Mor1	22	✓	—	—	—	—	—	—	—	✓	—	—	—	✓	✓	✓	—	—	—	—	—	—	—	**?**	✓
RBG_13_68_16	23	✓	✓	✓	✓	✓	✓	✓	✓	✓	—	—	—	—	—	—	—	—	—	—	—	—	—	—	**—**
13_1_20CM_2_68_14	U	—	✓	—	—	✓	—	—	—	✓	—	—	—	—	—	—	—	—	—	—	✓	—	—	—	**—**
13_1_40CM_2_68_10	U	—	✓	✓	✓	✓	✓	✓	✓	—	—	—	—	—	—	—	—	—	—	—	✓	—	—	—	**—**
13_1_40CM_2_68_5	U	—	✓	✓	—	✓	—	✓	—	—	✓	—	—	—	—	—	—	—	—	—	✓	—	—	—	**—**
13_1_40CM_4_69_4	U	—	—	—	—	—	—	✓	—	✓	—	—	—	—	—	—	—	—	—	—	—	—	—	—	**—**

*✓, Present; 2, two copies detected; 3, three copies detected; —, absent; ?, unclear; Meth., methylation; Put., putative; U, unknown; note that the absence of a gene may be caused by the incompleteness of the genome*.

a *Genbank accession see Tables S2–S7*.

The genes of the MEP pathway were (partially) identified in all the analyzed genomes considered here except for the genome classified within SD22 (Table 3). This genome, however, contained most genes of the mevalonate pathway for DMAPP biosynthesis (Zhao et al., 2013; Figure 7). All but one examined acidobacterial genomes also encoded *ispA*, the gene encoding farnesyl diphosphate synthase. Genes encoding enzymes involved in squalene synthesis from farnesyl diphosphate were only detected in genomes of SD1, SD2, SD6, and the SD1/3 cluster. In most of the genomes of SD2, the *hpnC* and *hpnD* are fused into one gene with approximately twice the size of the individual *hpnC* and *hpnD* genes. This occurs more often with genes that are of the same functional category (e.g., Yanai et al., 2001). Although the organization of the *hpnC*—*hpnD*—*hpnE* cluster is most common in bacteria (Pan et al., 2015), a BLAST search indicated that a fused *hpnCD* gene also occurs in other bacterial species; this SD2 gene is most closely related to the *hpnCD* gene in specific *Actinobacteria* (e.g., *Actinopolyspora* species, *Blastococcus saxobsidens*) and *Alphaproteobacteria* (e.g., *Rhodospirillum centenum*).

*Shc* and some other crucial genes for the biosynthesis of BHP (*hpnH, hpnG*, potentially *hpnA*) and its derivatives (*hpnI, hpnJ, hpnO*) were only identified in the environmental genomes of the SD1, SD2, and SD1/3 group (Table 3). The SD22 genome contains *shc* and *hpnH* but it lacks the genes for squalene synthesis from farnesyl diphosphate commonly encountered in *Acidobacteria*. However, a gene was identified that shows homology with genes annotated as squalene synthase in other bacterial groups. This tentatively suggests that the SD22 *Acidobacterium* is capable of BHP biosynthesis, albeit in a rather different way than all other acidobacteria examined. No genes involved in the methylation of BHPs were identified in the acidobacterial genomes obtained from the environmental genomes, including those that are phylogenetically closely related to “*Ca*. K. versatilis Ellin345.” Consequently, it seems likely that only acidobacteria falling in the cluster phylogenetically related to SD1, SD2 and SD3, and SD22 are potentially capable of BHP synthesis.

The genomic organization of the genes involved in BHP synthesis in the apparently most complete environmental genomes (i.e., based on the presence of an (almost) complete set of genes for BHP biosynthesis) is shown in Figure 7. The SD1 genomes, which are most closely related to “*Ca*. K. versatilis Ellin345,” show an organization that is comparable to most other SD1 acidobacteria. In the species of SD2 gene, cluster A is always encountered, albeit that the genes *hpnC* and *hpnD* are in most cases fused (Figure 7). Gene cluster C is in all cases the same but gene cluster B occurs in a fragmented form. SD3 and SD 1/3 acidobacteria contain gene cluster C but clusters A and B are reduced by the absence of *ug2* and *ug3*, respectively, which are commonly associated with these clusters in SD1 genomes. In *C. thermophilum* (SD4) and the environmental genome Mor1 (SD22) the organization of the BHP genes as observed in SD1 *Acidobacteria* is no longer evident.

## Discussion

### Relation of pheno- with genotype with respect to BHP production

In this study, we compare BHP and C_30_ hopene production with the biosynthetic capacity at the gene level for hopanoid production. Although 38 acidobacterial strains were analyzed for the presence of BPH production in culture and the 31 acidobacterial genomes were screened for the BHP biosynthetic pathway genes, this comparison is somewhat complicated by the fact that only for 15 strains both hopanoid production and genome data are available at the same time (Table 2; species in bold). However, the 16S rRNA gene phylogenetic tree shows that, in most cases, for strains that only have genome data available we have analyzed the BHP production in phylogenetically closely related strains (Figure 1).

The general picture that emerges from this comparison is that there is an excellent match in pheno- and genotype in terms of hopanoid production. All analyzed SD1 and SD3 acidobacterial strains have the genetic capacity for hopanoid production and do produce hopanoids. There are two out of 21 tested SD1 acidobacteria (*Terriglobus* sp. KMR, and *Telmatobacter bradus* TPB6017^T^; Table 1) that did not produce C_30_ hopenes. However, in these cases the production of BHPs was not determined. Examination of other strains showed that even when hop-17(21)-ene is absent, BHPs may still be produced (Table 1), indicating that the absence of hop-17(21)-ene cannot be interpreted to indicate the absence of hopanoid production. Furthermore, in both cases where hop-17(21)-ene was lacking, positive evidence for hopanoid production was obtained for phylogenetically closely related strains (i.e., *Terriglobus roseus* KBS63^T^, and *Telmatobacter* sp. 15–8A and 15–28; Table 1).

Also for SD4 acidobacteria a good match between pheno- and genotype was observed. Only *C. thermophilum* harbored the genes for hopanoid production (Table 2) and also did produce them (Table 1). The other SD4 acidobacteria did not produce hopanoids nor did possess the biosynthetic genes. It should be noted that *C. thermophilum* is quite distinct from other SD4 acidobacteria with respect to the 16S rRNA gene phylogeny (Figure 1), physiology (i.e., the only photoheterotroph in the phylum *Acidobacteria*; Bryant et al., 2007), and membrane lipid composition (Sinninghe Damsté et al., 2014), and this is confirmed here by its unique capacity of producing hopanoids as the only SD4 acidobacterium so far. In a study of the genome of *C. thermophilum* B, Costas et al. (2012a) already pointed out that this species is rather distinct from other *Acidobacteria*; in <20% the closest relative of a gene was a gene of another acidobacterium (i.e., *A. capsulatum*, “*Ca*. K. versatilis Ellin345,” and “*Ca*. S. usitatus Ellin 6076”), whereas this was the case for slightly over 50% for other *Acidobacteria*. This is also evident from our data: both the *shc* gene (Figure 5) and the second copy of the *hpnD* gene (Figure 6) are most closely related to other groups of bacteria. This once more illustrates the unique position of *C. thermophilum* within the *Acidobacteria*.

All other examined acidobacteria from other subdivisions (i.e., SD6, SD8, SD10, and SD23) did not produce hopanoids (Table 1) and also did not possess the genetic capacity to do so (Table 2). So this dataset allows concluding that (i) there is a clear dichotomy within the phylum *Acidobacteria* with respect to hopanoid production, and (ii) that when acidobacterial strains possess the genes to produce hopanoids, they express them, at least under the (highly variable) culture conditions that were used to grow them.

This conclusion is supported by the occurrence of genes involved in BHP biosynthesis in environmental acidobacterial genomes belonging to SD1 (Table 3). This survey, however, also suggested that BHP biosynthesis may also occur in some other SDs. The environmental genomes of SD2 (which has no cultured relatives) derived from soil (Butterfield et al., 2016) appeared to have the genetic potential to produce BHPs. This also holds true for a group of acidobacteria occurring in low-oxygen groundwater (Anantharaman et al., 2016), phylogenetically related to SD1 and SD3 acidobacteria. Lastly, the environmental genome of an SD22 acidobacterium associated with the cyanobacterium *Moorea producens* also shows distinct sign of the capability to produce BHPs, albeit in a completely different way than the other acidobacteria. Genomes of SD6 acidobacteria obtained from environmental metagenomes are genetically capable of producing squalene but do not possess *shc* and other BHP-related genes (Table 3), as observed for the SD6 acidobacteria available in pure culture (Table 2). The other genomes of acidobacteria obtained from metagenomes analyzed here do not show any signs of the genetic capacity to biosynthesize BHPs, in good agreement with the genomic data of acidobacteria available in culture (Table 2). This also confirms that *C. thermophilum* B and OC1 are an exception in SD4.

#### Squalene biosynthesis: a potential shortcut inherited from betaproteobacteria?

Pan et al. (2015) recently established that the production of squalene, an important intermediate in the production of hopanoids, in most bacteria proceeds through a three-step mechanism (Figure 4): HpnD catalyzes the formation of presqualene diphosphate (PSPP) from two molecules of farnesyl diphosphate (FPP), HpnC converts PSPP to hydroxysqualene (HSQ), and HpnE, a member of the amine oxidoreductase family, reduces HSQ to squalene. This was rigorously established by cloning and expressing the *hpnC, hpnD*, and *hpnE* genes from the hopanoid-producing bacteria *Z. mobilis* and *R. palustris* into *Escherichia coli*, a bacterium that does not contain genes homologous to *hpnC, hpnD*, and *hpnE*, and their functions were established *in vitro* and *in vivo*. These three genes occur typically concatenated in a cluster often directly followed by *shc* (*hpnF*), forming a four-gene cluster in the genome (Perzl et al., 1998).

Most of the hopanoid-producing cultivated acidobacteria possess *hpnC, hpnD*, and *hpnE*, commonly but not always (i.e., in 12 of 21 genomes) organized in a gene cluster (red box in Figure 7). However, this gene cluster is not directly located upstream of *shc*, as is commonly found in other bacteria (e.g., Perzl et al., 1998), but *hpnC* is often preceded by a gene with an unknown function (*ug2*), often annotated as coding for a Zn-binding alcohol dehydrogenase composed of ca. 350 amino acids. This gene is present in all SD1 and SD3 acidobacteria but not in *C. thermophilum*, suggesting that it is not essential for squalene biosynthesis but somehow is associated with *hpnC, hpnD*, and *hpnE*. The dispersal of the *hpnC, hpnD*, and *hpnE* genes in some other acidobacteria genomes (e.g., *Bryobacter aggregatus* MPL3^T^; Figure 7) indicates that organization of these genes in a gene cluster is also not essential for squalene production.

Interestingly, four cultivated acidobacterial species do not possess all three genes commonly used for conversion of FPP into squalene (*hpnC, hpnD*, and *hpnE*) but lack *hpnE* (i.e., *Acidobacteriaceae* sp. KBS89) or both *hpnC* and *hpnE* (i.e., *S. bohemicum* S15, *G. mallensis* MP5ACTX8, *Acidobacteriaceae* sp. TAA66). Unfortunately, these strains were not tested for hopanoid production. However, phylogenetically closely related strains were (Figure 1), suggesting that they still would be able to produce hopanoids and, thus, squalene. Remarkably, in these strains, and in *E. aggregans*, a gene annotated as coding for farnesyl diphosphate farnesyl transferase (*fdfT*) was detected, but was absent in any of the other acidobacteria. This gene is somewhat comparable to the gene encoding for squalene synthase, an enzyme occurring in many eukaryotes, where it catalyzes two reactions, i.e., the coupling of two molecules of FPP to give PSPP and the subsequent NADPH-dependent reductive rearrangement of PSPP to squalene without the release PSPP from the active site. A similar enzyme has been characterized in a cyanobacterium (Lee and Poulter, 2008) and a gammaproteobacterium (Ohtake et al., 2014). The FdfT enzymes in the acidobacteria show a sequence identity of ca. 30% with the characterized bacterial squalene synthases but the protein alignments (Figure S1) show that the active sites of the enzymes are more conserved, suggesting that the detected *fdfT* genes encode an enzyme capable of direct conversion of two molecules of farnesyl diphosphate into squalene. The presence of both *hpnD* and *fdfT* in the acidobacteria that lack the *hpnC*-*hpnD*-*hpnE* cassette may alternatively suggest that *fdfT* encodes an enzyme that converts PSPP directly into squalene (Figure 4) but this hypothesis needs to be tested.

Remarkably, the *fdfT* occurring in five acidobacterial species is phylogenetically closely related to *fdfT* of several *Parabulkholderia* and *Caballeronia* species (*Betaproteobacteria*; Figure 6); the sequence identity at the protein level is ca. 70–80%. This high level of sequence similarity suggests that lateral gene transfer has introduced this gene. Interestingly, some of the closely related species were isolated from soil (e.g., *Caballeronia glathei*, Zolg and Ottow, 1975; *Paraburkholderia sartisoli*, Vanlaere et al., 2008) and decaying wood (e.g., *P. sordidicola*; Lim et al., 2003), similar ecological niches as occupied by SD1 *Acidobacteria*, supporting the suggestion of horizontal gene transfer. Naumoff and Dedysh (2012) have previously recognized horizontal gene transfer between *Bacteroidetes* and *Acidobacteria* for genes encoding alfa-L-rhamnosidases and proposed that sharing a similar ecological niche facilitated such events. *E. aggregans* possesses even two copies of the *fdfT* gene in addition to the complete gene cluster A (Table 2). This is also one of the SD1 *Acidobacteria* with the largest genome size (8.2 Mb; Table 2), which perhaps explains why it is carrying genes that seem not essential. Challacombe et al. (2011) suggested earlier for “*Ca*. S. usitatus Ellin6076” that its relatively large genome (9.9.Mb; 2–5 times as large as most other *Acidobacteria*) has arisen by horizontal gene transfer and widespread small-scale gene duplications, resulting in an increased number of paralogs.

#### BHP biosynthesis

The synthesis of (composite) BHPs from squalene in bacteria is performed by a sequence of enzymatic reactions (Figure 3) for which quite a number of coding genes is known (Table S1). For the acidobacterial genomes studied, it is apparent that those that possess *shc* also contain *hpnH* and *hpnG* (Table 2), the genes encoding enzymes for the first two steps of the biosynthesis of BHT. The phylogeny of these proteins (like *shc*; Figure 5) is broadly similar to that of the 16S rRNA gene, suggesting that these genes are inherited from a common ancestor. In all SD1 *Acidobacteria* (except for “*Ca*. K. versatilis Ellin345”) *hpnH* forms together with *shc* and *ispH* a gene cluster. “*Ca*. K. versatilis Ellin345” is also with respect to 16S rRNA gene phylogeny (Figure 1) the most remote species. In all SD1 and SD3 *Acidobacteria, hpnG* forms a gene cluster with *hpnA*, which is present in all acidobacteria that possess *shc, hpnH*, and *hpnG*, suggesting that it is essential for the formation of BHPs. It is annotated as a hopanoid-associated sugar epimerase. It may catalyze the step transformation of ribosyl hopanes to BHT (Figure 4) for which the enzyme is still unknown. However, Schmerk et al. (2015) presented some evidence that *hpnA* was not essential for BHT synthesis in *Burkholderia cenocepacia* and some BHT producers (like *Rhodopseudomonas*) do not have a copy of *hpnA* in their genome (Welander et al., 2012).

BHT can subsequently be converted into composite BHPs such as BHT cyclitol ether (Figure 4). For this latter conversion three genes are required: *hpnI, hpnK*, and *hpnJ* (Table S1). The only SD4 acidobacterium capable of producing BHPs, *C. thermophilum* B, is also the only acidobacterium that possesses all these genes (Table 2). In good agreement with this, intact BHP analysis of *C. thermophilum* B has revealed the biosynthesis of BHT cyclitol ether, in addition to BHT (Costas et al., 2012b). In contrast, the SD1 and SD3 Acidobacteria do possess *hpnJ* and *hpnI* but lack *hpnK* (Table 1), suggesting that they are not able to produce BHT cyclitol ether. Since the Rohmer degradation method transforms both BHT and its cyclitol ether derivative into homohopanol, our dataset does not allow confirming this point.

The *hpnO* gene, encoding the enzyme responsible for the last step in the formation of amino bacteriohopanetriol from BHT (Figure 4) was detected in SD1 (but not all) and SD3 acidobacterial genomes, suggesting that these bacteria would be capable of producing amino bacteriohopanetriol.

#### Methylation of hopanoids in acidobacteria

Welander et al. (2010) and Welander and Summons (2012) previously reported the presence of the genes encoding the enzymes responsible for methylation of the A-ring of biohopanoids (*hpnP* and *hpnR*) in the genome of the acidobacterium “*Ca*. K. versatilis Ellin345.” Methylated BHPs were indeed detected in cultures of “*Ca*. K. versatilis Ellin345” in our study although the relative amounts were low and highly variable. Surprisingly, the Rohmer degradation products of the BHPs of “*Ca*. K. versatilis Ellin345” contain a tentatively identified dimethylated homohopanol. To the best of our knowledge, hopanoids methylated at both position 2 and 3 have not been previously encountered. However, its presence is consistent with the presence of both the *hpnP* and *hpnR* methylation genes, which is very rare in the bacterial domain. The only other bacterial genome in the NCBI database that possesses these two genes is *Methylobacterium nodulans* ORS2060 (Marx et al., 2012), a soil bacterium growing on C_1_ compounds belonging to the *Alphaproteobacteria* (Jourand et al., 2004). The dimethylated BHPs only occurred in one of the three batch cultures of “*Ca*. K. versatilis Ellin345” examined. We tentatively attribute this to the growth phase in which the culture was harvested. It has been demonstrated for *M. capsulatus* that 3-methylhopanoids appear to be required for the maintenance of intracytoplasmic membranes and cell survival in late stationary phase (Welander and Summons, 2012). Consequently, methylated BHPs may be primarily formed in this stage of the growth curve but this will require further research.

Remarkably, only one of the many investigated acidobacterial species produces or has potential genetic capacity to produce methylated BHPs. For the acidobacterial environmental genomes studied also no evidence for production of methylated BHPs was obtained, even though some genomes were closely related to “*Ca*. K. versatilis Ellin345” (Figure 1). This has been observed for other bacterial phyla; Welander and Summons (2012) reported that only 3 out of 38 *Burkholderia*, 8 out of 43 *Streptomyces*, and 1 out 8 *Methylobacterium* genomes harbored the *hpnR* gene, while all possessed the *shc* gene. Also for the *hpnP* gene it has been observed that only a limited number of species of a bacterial phylum (e.g., *Cyanobacteria*) possess this gene (Welander et al., 2010). This poses questions on the evolutionary origin of these methylases. For the *Acidobacteria* it seems rather unlikely that their common ancestor possessed both genes and all but one of the examined species (i.e., “*Ca*. K. versatilis Ellin345”) lost them. It seems, therefore, much more plausible that “*Ca*. K. versatilis Ellin345” obtained these genes through horizontal gene transfer, although both *hpnP* and *hpnR* gene of “*Ca*. K. versatilis Ellin345” are not closely related to any other in other bacterial phyla, a situation that is clearly different from the case observed for the *fdfT* gene (see above and Figure 6). Ricci et al. (2015) concluded that the *hpnP* gene originated in a subset of the *Alphaproteobacteria* and that it was likely horizontally transferred into the *Cyanobacteria* after their major divergences, providing further evidence that the ancestral function of 2-methylhopanoids was not related to oxygenic photosynthesis (*cf*. Summons et al., 1999). They also concluded from their analysis that at approximately the same time as the horizontal *hpnP* gene transfer event between *Alphaproteobacteria* and *Cyanobacteria, hpnP* appears to have been laterally transferred to the *Acidobacteria*. This ancient horizontal gene transfer would explain why the *hpnP* gene of “*Ca*. K. versatilis Ellin345” is not closely related to any other *hpnP* gene in other bacterial phyla. However, it remains enigmatic why “*Ca*. K. versatilis Ellin345” is the only acidobacterium carrying this gene, a situation, which is clearly distinct from that in the *Cyanobacteria* where many species possess the *hpnP* gene (Ricci et al., 2015).

### Implications for BHPs in the environment

BHPs are omnipresent biomarkers that are being used for a wide variety of applications. Environmental microbiology studies using 16S rRNA or functional genes (e.g., *shc* and *hpnR*) have been applied to trace potential sources of BHPs in the environment (e.g., Pearson et al., 2007; Coolen et al., 2008; Ricci et al., 2014). However, culture studies remain essential for a proper understanding of sources of BHPs in the environment. This study provides for the first time a comprehensive overview of the occurrence of BHPs in *Acidobacteria*. Our results indicate that, in the phylogenetic groups investigated, BHP biosynthesis is mostly limited to SDs 1, 2, and 3 and a closely affiliated group (SD1/3), out of the many SDs of the phylum. This may suggest that in the environment *Acidobacteria* may not be considered as important sources of BHPs. However, many studies have indicated that SD1, 2, and 3 *Acidobacteria* are among the most abundant acidobacterial groups in wetlands and soil, whereas SD1 members thrive in lakes (see Kielak et al., 2016 for a recent review). Furthermore, *Acidobacteria* often form an important fraction of the bacterial community in soils and peat bogs (e.g., Chan et al., 2006; Weijers et al., 2009; i.e., up to 80%). Therefore, in a wide variety of environments acidobacteria could, despite the somewhat restricted BHP biosynthetic capacity in the phylum as a whole, still be considered potentially quantitatively important BHP producers in the environment.

Methylated BHPs are considered to be even better constrained biomarkers in the environment, although this has been challenged by genomic (Welander et al., 2010; Welander and Summons, 2012; Ricci et al., 2015) and environmental microbiological (Ricci et al., 2014) studies. These authors also discovered the methylation genes of the BHP pathway in “*Ca*. K. versatilis Ellin345” and this study confirmed that this species under certain conditions produces small amounts of a dimethylated BHP. However, “*Ca*. K. versatilis Ellin345” is the only acidobacterium identified so far that is able to produce methylated hopanoids and, therefore, *Acidobacteria* are unlikely to play an important role in sourcing methylated BHPs in the environment.

## Author contributions

JD and LV generated hypothesis and planned experiments. LV, SD, and BF have grown cultures. WR performed lipid analyses and JD and WR interpreted the data. JD and LV analyzed genome data, interpreted the data and wrote the paper. All other authors provided comments on the text.

### Conflict of interest statement

The authors declare that the research was conducted in the absence of any commercial or financial relationships that could be construed as a potential conflict of interest.
