# Supplementary material for: Pheno- and Genotyping of Hopanoid Production in Acidobacteria
Source: Front Microbiol. 2017 Jun 8;8:968. doi: 10.3389/fmicb.2017.00968 (PMC5462960; doi:10.3389/fmicb.2017.00968)
Supplement: Supplementary file 1 [file DataSheet1.pdf]

Supplementary Material

Pheno- And Genotyping Of Hopanoid Production In Acidobacteria

Jaap S. Sinninghe Damsté *, W. Irene C. Rijpstra, Svetlana Dedysh, Bärbel U. Foesel,

and Laura Villanueva

*** Correspondence:** Corresponding Author: damste@nioz.nl

1 Supplementary Figure

2 Supplementary Tables

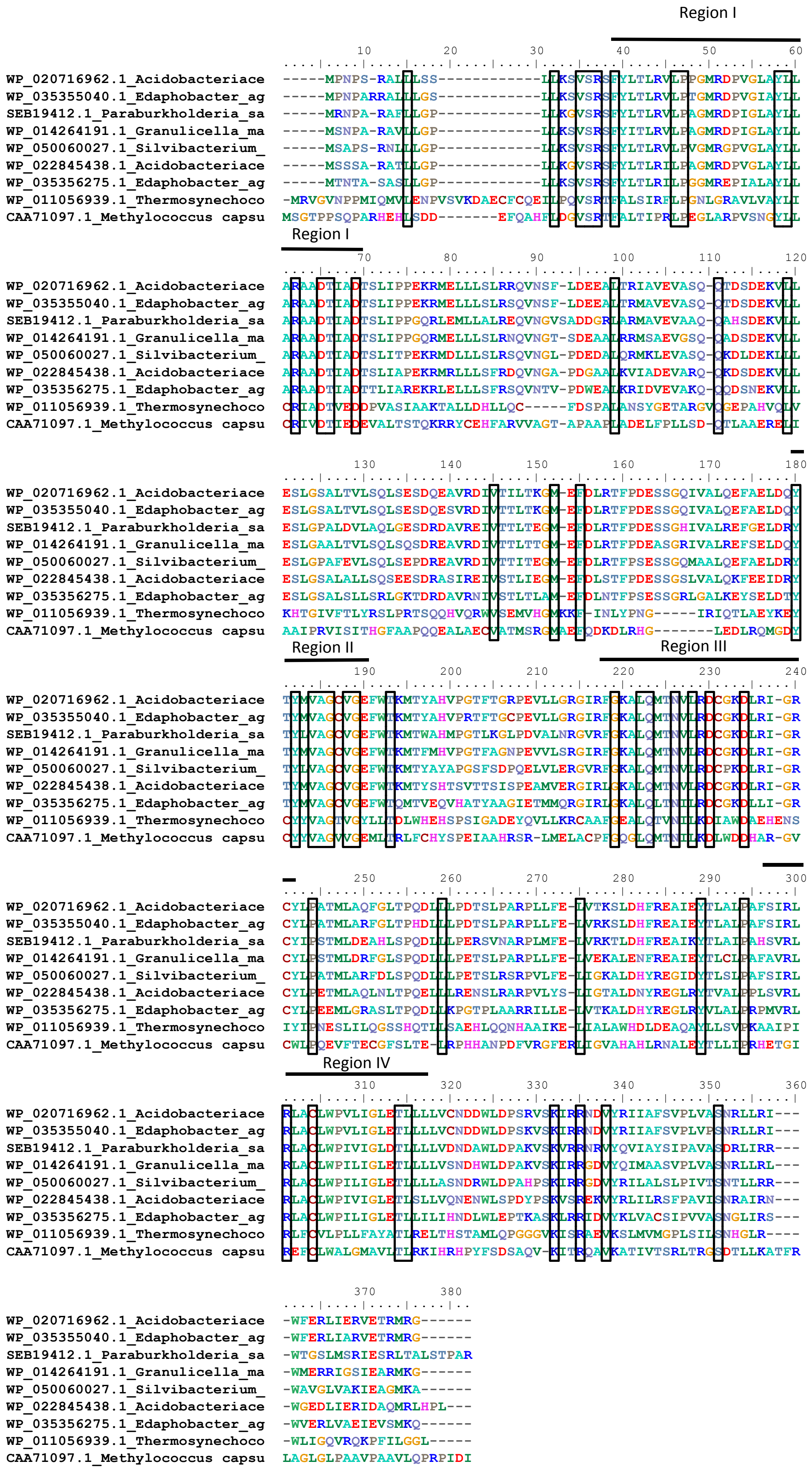

Figure S1: Amino acid sequence alignments for FdtP for *Silvibacterium bohemicum* S15 (WP_050060027.1), Acidobacteriaceae bacterium KBS 89 (WP_020716962.1), *Edaphobacter aggregans Wbg-1* (WP_035355040.1, WP_035356275.1), *Granulicella mallensis* MP5ACTX8 (WP_014264191.1), Acidobacteriaceae bacterium TAA166 (WP_022845438.1), *Parabulkholderia sartisoli* (SEB19412.1) in comparison to those of *Thermosynechococcus elongates* BP-1 (WP_011056939) (Lee and Poulter, 2008) and *Methylococcus capsulatus* ATCC 33009 (CAA71097.1). Dashes indicate gaps that were introduced to maximize the allignments. The conserved regions I-IV, previously identified on basis of comparison with eukaryotic squalene synthases ((Lee and Poulter, 2008) are indicated.

Table S1: Genes involved in the biosynthesis of BHPs and some other unknown genes often closely located to these genes in genomes of *Acidobacteria*.

Name	Annotation	Function of protein	Reference
dxs	1-deoxy-D-xylulose-5-phosphate synthase	Catalyzes the condensation of pyruvate and glyceraldehyde 3-phosphate to yield 1-deoxy-D-xylulose-5-phosphate (DXP)	Zhao et al. (2013)
dxr	1-deoxy-D-xylulose 5-phosphate reductoisomerase	Catalyzes the NADP-dependent rearrangement and reduction of DXP to 2-C-methyl-D-erythritol 4-phosphate (MEP)	Zhao et al. (2013)
ispD	2-C-methyl-D-erythritol 4-phosphate cytidylyltransferase	Catalyzes the formation of 4-diphosphocytidyl-2-C-methyl-D-erythritol from CTP and ME.	Zhao et al. (2013)
ispE	4-diphosphocytidyl-2-C-methyl-D-erythritol kinase	Catalyzes the phosphorylation of the position 2 hydroxy group of 4-diphosphocytidyl-2-C-methyl-D-erythritol	Zhao et al. (2013)
ispF	2-C-methyl-D-erythritol 2,4-cyclodiphosphate synthase	Catalyzes the conversion of 4-diphosphocytidyl-2-C-methyl-D-erythritol 2-phosphate (CDP-ME2P) to 2-C-methyl-D-erythritol 2,4-cyclodiphosphate (ME-CPP)	Zhao et al. (2013)
ispG	4-hydroxy-3-methylbut-2-en-1-yl diphosphate synthase	Converts 2-C-methyl-D-erythritol 2,4-cyclodiphosphate (ME-2,4cPP) into 1-hydroxy-2-methyl-2-(E)-butenyl 4-diphosphate, using flavodoxin as the reducing agent	Zhao et al. (2013)
ispH	4-hydroxy-3-methylbut-2-en-1-yl diphosphate reductase	Converts 1-hydroxy-2-methyl-2-(E)-butenyl 4-diphosphate into isopentenyl diphosphate (IPP) and dimethylallyl diphosphate (DMAPP)	Zhao et al. (2013)
orfD	Isopentenyl-diphosphate delta-isomerase type II	Catalyzes the 1,3-allylic rearrangement of IPP to DMAPP	Kaneda et al. (2001) PNAS 98(3): 932–937
hmgcR	3-hydroxy-3-methyl-glutaryl-coenzyme A reductase	Catalyzes the conversion of 3-hydroxy-3-methyl-glutaryl-coenzyme A to mevalonic acid in the mevalonate pathway	Miziorki (2011)
mvK	Mevalonate kinase	Catalyzes the conversion of mevalonic acid in mevalonate-5-phosphate	Miziorki (2011)
mvD	Mevalonate diphosphate decarboxylase	Catalyzes the conversion of mevalonate pyrophosphate into isopentenyl pyrophosphate	Miziorki (2011)
ispA	Farnesyl diphosphate synthase	Production of farnesyl diphosphate from IPP and DMAPP	
hpnD	Squalene/phytoene synthase	Production of presqualene diphosphate from farnesyl diphosphate	Pan et al. (2015)
hpnC	Squalene/phytoene synthase	Conversion of presqualene diphosphate into hydroxysqualene	Pan et al. (2015)
hpnE	Amine oxidoreductase	Reduction of hydroxysqualene	Pan et al. (2015)
fdfT	Farnesyl diphosphate farnesyl transferase	Putative enzyme for producing squalene from farnesyl diphosphate	
shc	Squalene hopene cyclase	Formation of diploptene from cyclization of squalene	Reipen et al. (1995)
hpnH	Hopanoid-associated radical SAM superfamily protein	Addition of adenosine to hopane skeleton	Bradley et al. (2010); Welander et al. (2012); Schmerk et al. (2015)
hpnG	Hopanoid-associated nucleosidase	Removal of adenine from adenosylhopane	Bradley et al. (2010); Welander et al. (2012); Schmerk et al. (2015)
hpnI	Hopanoid-associated glycosyl transferase	Transfer of acetylglucosamine from UDP-acetylglucosamine to BHT	Schmerk et al. (2015)
hpnK	Hopanoid biosynthesis associated protein	Deacetylation of BHT acetylglucosamine	Schmerk et al. (2015)
hpnJ	Hopanoid-associated radical SAM superfamily protein	Ring contraction to generate BHT cyclitol ether	Schmerk et al. (2015)
hpnO	Aminotransferase	Introduces the amino group to produce aminobacteriohopanetriol	Welander et al. (2012)
hpnP	Hopanoid C-2 methylase HpnR	Introduces a methyl group at position C-2 in a BHP or C_{30} hopanoid	Welander et al. (2010)
hpnR	Hopanoid C-3 methylase HpnR	Introduces a methyl group at position C-3 in a BHP or C_{30} hopanoid	Welander and Summons (2012)

hpnA	Hopanoid-associated sugar epimerase	Not established	
hpnB	Hopanoid-associated glycosyl transferase	Not established	
ug1	Zn-dependent alcohol dehydrogenase (ca. 370 AA)	Not established	
ug2	Zn-binding alcohol dehydrogenase (ca. 350 AA)	Not established	
ug3	Hypothetical protein (ca. 150 AA)	Not established	
ug4	Hypothetical protein (ca. 130 AA)	Not established	
ug5	Transporter (ca. 140 AA)	Not established	

Table S2: Presence of the *ispA* gene encoding farnesyldiphosphate synthase in genomes of cultivated *Acidobacteria* and environmental genomes based on BLAST searches of the protein sequence in the NCBI database.

***Acidobacterial* genome**	**SD**	***ispA***	**SI (%)**	**AC**
**Cultures**				
Acidobacterium capsulatum 161	1	✓	100	WP_015896781.1
Acidobacterium ailaaui PMMR2	1	✓	81	WP_026443680.1
Silvibacterium bohemicum S15	1	✓	79	WP_050058068.1
Acidobacteriaceae bacterium KBS 83	1	✓	70	WP_026335820.1
Acidobacteriaceae bacterium KBS 89	1	✓	59	WP_020712160.1
Terracidiphilus gabretensis S55	1	✓	70	WP_058189358.1
Edaphobacter aggregans Wbg-1	1	✓	66	WP_035351326.1
Acidobacteriaceae bacterium URHE0068	1	✓	77	WP_026444623.1
Acidobacteria bacterium KBS 146	1	✓	66	WP_026386959.1
Terriglobus saanensis SP1PR4	1	✓	68	WP_013569474.1
Granulicella mallensis MP5ACTX8	1	✓	64	WP_014267017.1
Granulicella tundricola MP5ACTX9	1	✓	63	WP_013579430.1
Granulicella pectinivorans TPB6011	1	✓	62	SFS13509.1
Bryocella elongata SN10	1	✓	60	SEG01803.1
Terriglobus roseus DSM 18391	1	✓	65	WP_014787013.1
Terriglobus sp. TAA 43	1	✓	67	WP_047498047.1
Terriglobus roseus GAS232	1	-		
Terriglobus roseus AB35.6	1	✓	65	SEC27148.1
Acidobacteriaceae bacterium TAA166	1	✓	66	WP_022844472.1
'*Ca.* Koribacter versatilis Ellin345'	1	✓	62	ABF42433.1
Acidobacteriaceae bacterium KBS 96	3	✓	53	WP_020721398.1
'*Ca.* Solibacter usitatus Ellin6076'	3	✓	54	ABJ82108.1
Bryobacter aggregatus MPL3	3	✓	51	WP_031499979.1
Chloracidobacterium thermophilum B	4	✓	50	WP_014100740.1
Chloracidobacterium thermophilum OC1	4	✓	50	WP_058867867.1
Pyrinomonas methylaliphatogenes K22	4	✓	44	WP_060635749.1
Luteitalea pratensis HEG_-6_39	6	✓	42	AMY09642.1
Holophaga foetida TMBS4	8	✓	43	WP_005036318.1
Geothrix fermentans H-5	8	✓	45	WP_026852490.1
Thermotomaculum hydrothermale AC55	10	✓	nd	ORF TTHT_0896
Thermoanaerobaculum aquaticum MP-01	23	✓	46	WP_038049832.1
**Environmental genomes**				
Acidobacteriales bacterium 59-55	1	✓	64	OJV41243.1
Acidobacteriales bacterium 13_1_20CM_4_56_7	1	–		
Acidobacteriales bacterium 13_1_40CM_3_55_5	1	✓	61	OLD16780.1
Acidobacteriales bacterium 13_2_20CM_2_55_5	1	✓	61	OLB88925.1
Acidobacteriales bacterium 13_2_20CM_55_8	1	✓	62	OLB23326.1
Acidobacteria bacterium 13_1_20CM_2_60_10	2	✓	53	OLE84963.1
Acidobacteria bacterium 13_1_20CM_3_58_11	2	✓	54	OLE48756.1

Acidobacteria bacterium 13_1_20CM_58_21	2	✓	54	OLD79709.1
Acidobacteria bacterium 13_1_20CM_4_57_6	2	–		
Acidobacteria bacterium 13_1_40CM_4_58_4	2	✓	53	OLC90121.1
Acidobacteria bacterium 13_1_40CM_4_61_5	2	–		
Acidobacteria bacterium 13_2_20CM_2_57_6	2	–		
Acidobacteria bacterium 13_2_20CM_57_17	2	✓	54	OLB40006.1
Acidobacteria bacterium 13_2_20CM_57_7	2	✓	52	OLB17076.1
Acidobacteria bacterium 13_2_20CM_58_27	2	✓	53	OLB25819.1
Acidobacteria bacterium 13_1_20CM_56_16	2'	✓	44	OLC30141.1
Acidobacteria bacterium RIFCSPLOWO2_02_FULL_59_13	1/3	✓	49	OFW23766.1
Acidobacteria bacterium RIFCSPLOWO2_02_FULL_61_28	1/3	✓	48	OFW00007.1
Acidobacteria bacterium RIFCSPLOWO2_12_FULL_54_10	1/3	✓	48	OFV99601.1
Acidobacteria bacterium RIFCSPLOWO2_12_FULL_59_11	1/3	✓	48	OFW07476.1
Acidobacteria bacterium RIFCSPLOWO2_12_FULL_60_22	1/3	✓	52	OFW38722.1
Acidobacteria bacterium RIFCSPHIGHO2_02_FULL_67_57	3/4	✓	53	OFV84683.1
Acidobacteria bacterium RIFCSPHIGHO2_12_FULL_67_30	3/4	✓	52	OFV88534.1
Acidobacteria bacterium OLB17	4	✓	46	KXK04241.1
Acidobacteria bacterium 13_1_20CM_3_53_8	4	✓	47	OLE51688.1
Acidobacteria bacterium RBG_16_70_10	4'	✓	47	OFV86782.1
Acidobacteria bacterium RIFCSPLOWO2_02_FULL_64_15	6	✓	40	OFV94460.1
Acidobacteria bacterium RIFCSPLOWO2_02_FULL_65_29	6	✓	40	OFW19478.1
Acidobacteria bacterium RIFCSPLOWO2_02_FULL_67_36	6	✓	44	OFW06494.1
Acidobacteria bacterium RIFCSPLOWO2_02_FULL_67_21	6	✓	41	OFW14569.1
Acidobacteria bacterium RIFCSPLOWO2_02_FULL_68_18	6	✓	40	OFW01586.1
Acidobacteria bacterium RIFCSPLOWO2_12_FULL_67_14b	6	✓	41	OFW43465.1
Acidobacteria bacterium 13_1_20CM_2_65_9	6	✓	42	OLE82011.1
Acidobacteria bacterium 13_1_40CM_2_64_6	6	✓	42	OLD54426.1
Acidobacteria bacterium 13_1_40CM_65_14	6	✓	42	OLC51735.1
Acidobacteria bacterium SCN 69-37	6	✓	39	ODS51911.1
Acidobacteria bacterium Mor-1	22	✓	47	ANM28771.1
Acidobacteria bacterium RBG_13_68_16	23	✓	45	OFV82917.1
Acidobacteria bacterium 13_1_20CM_2_68_14	U	✓	51	OLE65119.1
Acidobacteria bacterium 13_1_40CM_2_68_10	U	-		
Acidobacteria bacterium 13_1_40CM_2_68_5	U	-		
Acidobacteria bacterium 13_1_40CM_4_69_4	U	✓	48	OLC54462.1

Key: SD = subdivision, ✓ = present, - = absent, SI = similarity (%) as blasted against the corresponding gene of *A. capsulatum* ATCC 51196, AC = Accession number in NCBI database, U = unknown

Table S3: Presence of the *hpnC, hpnD, hpnE* and putative *fdpT* genes encoding enzymes for the synthesis of squalene in *Acidobacteria*l genomes of cultures and environmental metagenomes based on BLAST searches of the protein sequence in the NCBI database.

***Acidobacterial* genome**	**SD**	***hpnC***	**SI[b]**	**AN**	***hpnD***	**SI[b]**	**AN**	***hpnE***	**SI[b]**	**AN**	***fdpT***	**SI[b]**	**AN**
**Culture**													
Acidobacterium capsulatum 161	1	✓		WP_015895982.1	✓		WP_015895981.1	✓		WP_015895980.1	-		
Acidobacterium ailaaui PMMR2	1	✓	78	WP_026441648.1	✓	69	WP_026441649.1	✓	60	WP_026441650.1	-		
Silvibacterium bohemicum S15	1	-			✓	68	WP_050062226.1	-			✓	100	WP_050060027.1
Acidobacteriaceae bacterium KBS 83	1	✓	69	WP_020710815.1	✓	59	WP_020710814.1	✓	61	WP_020710813.1	-		
Acidobacteriaceae bacterium KBS 89	1	✓	55	WP_020713414.1	✓	60	WP_020713415.1	✓			✓	75	WP_020716962.1
Terracidiphilus gabretensis S55	1	✓	70	WP_058185580.1	✓	63	WP_058185519.1	✓	52	WP_058185520.1	-		
Edaphobacter aggregans Wbg-1	1	✓	53	WP_035350376.1	✓	58	WP_035350375.1	✓	50	WP_035350374.1	✓	75<br>64	WP_035355040.1<br>WP_035356275.1
Acidobacteriaceae bacterium URHE0068	1	✓	71	WP_051566372.1	✓	62	WP_026448080.1	✓	53	WP_051566374.1	-		
Acidobacteria bacterium KBS 146	1	✓	51	WP_026386235.1	✓	61	WP_026386234.1	✓	50	WP_026386233.1	-		
Terriglobus saanensis SP1PR4	1	✓	55	WP_013568394.1	✓	53	WP_013568395.1	✓	46	WP_013568396.1	-		
Granulicella mallensis MP5ACTX8	1	-			✓	51	WP_014265798.1	-			✓	74	WP_014264191.1
Granulicella tundricola MP5ACTX9	1	✓	55	WP_013580011.1	✓	56	WP_013580012.1	✓	47	WP_013580013.1	-		
Granulicella pectinivorans TPB6011	1	✓	53	SFS16941.1	✓	55	SFS16936.1	✓	48	SFS16932.1	-		
Bryocella elongata SN10	1	✓	50	SEF64291.1	✓	54	SEF64330.1	✓	47	SEF64359.1	-		
Terriglobus roseus DSM 18391	1	✓	51	WP_014785728.1	✓	50	WP_014785729.1	✓	48	WP_014785730.1	-		
Terriglobus sp. TAA 43	1	✓	54	WP_047487867.1	✓	47	WP_047487864.1	✓	49	WP_047487859.1	-		
Terriglobus roseus GAS232	1	✓	54	SDF10618.1	✓	47	SDF10652.1	✓	49	SDF10676.1	-		
Terriglobus roseus AB35.6	1	✓	50	SEB36906.1	✓	52	SEB36915.1	✓	48	SEB36925.1	-		
Acidobacteriaceae bacterium TAA166	1	-			✓	62	WP_026392933.1	-			✓	67	WP_022845438.1
'*Ca.* Koribacter versatilis Ellin345'	1	✓	64	WP_011522548.1	✓	51	WP_011522549.1	✓	55	WP_011522550.1	-		
Acidobacteriaceae bacterium KBS 96	3	✓	52	WP_020719488.1	✓	44	WP_020719487.1	✓	49	WP_020719725.1	-		
'*Ca.* Solibacter usitatus Ellin6076'	3	✓	53	WP_011683035.1	✓	44	WP_011683036.1	✓	48	WP_011685297.1	-		
Bryobacter aggregatus MPL3	3	-			✓	41	WP_035957817.1	✓	44	WP_035957682.1	-		
Chloracidobacterium thermophilum B	4	✓	44	AEP13731.1	✓	38<br>40	WP_014101470.1<br>WP_014099727.1	✓	28	WP_014100595.1	-		
Chloracidobacterium thermophilum OC1	4	✓[b]			✓	38<br>40	WP_058865892.1<br>WP_058867469.1	✓	27	WP_058867994.1	-		
Pyrinomonas methylaliphatogenes K22	4	-			-			-			-		
Luteitalea pratensis HEG_-6_39	6	✓	35	AMY12733.1	✓	38	AMY12732.1	✓	32	AMY12731.1	-		
Holophaga foetida TMBS4	8	-			-			-			-		
Geothrix fermentans H-5	8	-			-			-			-		
Thermotomaculum hydrothermale AC55	10	-			-			-			-		
Thermoanaerobaculum aquaticum MP-01	23	-			-			-			-		
**Environmental genomes**													
Acidobacteria bacterium 59-55	1	✓	52	OJV42131.1	✓	57	OJV41583.1	✓	47	OJV41582.1	-		
Acidobacteriales bacterium 13_1_20CM_4_56_7	1	-			-			-			-		

Acidobacteriales bacterium 13_1_40CM_3_55_5	1	✓	62	OLD18787.1	✓	56	OLD18773.1	✓	55	OLD18774.1	-		
Acidobacteriales bacterium 13_2_20CM_2_55_5	1	✓	62	OLB87678.1	✓	55	OLB87673.1	✓	57	OLB87672.1	-		
Acidobacteriales bacterium 13_2_20CM_55_8	1	✓	63	OLB22764.1	✓	56	OLB22763.1	✓	58	OLB22762.1	-		
Acidobacteria bacterium 13_1_20CM_2_60_10	2	✓[a]	53	OLE83119.1	✓[a]	41	OLE83119.1	✓	43	OLE83120.1	-		
Acidobacteria bacterium 13_1_20CM_3_58_11	2	-			-			-			-		
Acidobacteria bacterium 13_1_20CM_58_21	2	✓[a]	52	OLB40690.1	✓[a]	40	OLB40690.1	✓	43	OLD83138.1	-		
Acidobacteria bacterium 13_1_40CM_4_57_6	2	-			-			-			-		
Acidobacteria bacterium 13_1_40CM_4_58_4	2	✓[a]	50	OLC96287.1	✓[a]	41<br>46	OLC96287.1<br>OLC90952.1	✓	41	OLC96288.1	-		
Acidobacteria bacterium 13_1_40CM_4_61_5	2	✓[a]	53	OLC88152.1	✓[a]	39	OLC88152.1	✓	43	OLC88151.1	-		
Acidobacteria bacterium 13_2_20CM_2_57_6	2	✓[a]	52	OLB88656.1	✓[a]	39	OLB88656.1	✓	39	OLB88667.1	-		
Acidobacteria bacterium 13_2_20CM_57_17	2	✓[a]	52	OLB40690.1	✓[a]	39	OLB40690.1	✓	43	OLB40689.1	-		
Acidobacteria bacterium 13_2_20CM_57_7	2	✓[a]	53	OLB21794.1	✓[ab]			-			-		
Acidobacteria bacterium 13_2_20CM_58_27	2	✓[a]	52	OLB28866.1	✓[a]	40	OLB28866.1	✓	43	OLB28865.1	-		
Acidobacteria bacterium 13_1_40CM_56_16	2'	✓	53	OLC39076.1	✓	42	OLC39075.1	✓	41	OLC39074.1	-		
Acidobacteria bacterium RIFCSPLOWO2_02_FULL_59_13	1/3	✓	55	OFW14230.1	✓	40	OFW14228.1				-		
Acidobacteria bacterium RIFCSPLOWO2_02_FULL_61_28	1/3	✓[b]			✓	41	OFV94765.1	✓	43	OFV94773.1	-		
Acidobacteria bacterium RIFCSPLOWO2_12_FULL_54_10	1/3	-			-			✓	41	OFV96917.1	-		
Acidobacteria bacterium RIFCSPLOWO2_12_FULL_59_11	1/3	-			-						-		
Acidobacteria bacterium RIFCSPLOWO2_12_FULL_60_22	1/3	✓	60	OFW33133.1	✓	43	OFW33134.1	✓	44	OFW33135.1	-		
Acidobacteria bacterium RIFCSPHIGHO2_02_FULL_67_57	3/4	-			-			-			-		
Acidobacteria bacterium RIFCSPHIGHO2_12_FULL_67_30	3/4	-			-			-			-		
Acidobacteria bacterium OLB17	4	-			-			-			-		
Acidobacteria bacterium 13_1_20CM_3_53_8	4	-			-			-			-		
Acidobacteria bacterium RBG_16_70_10	4'	-			-			-			-		
Acidobacteria bacterium RIFCSPLOWO2_02_FULL_64_15	6	✓	35	OFV91905.1	✓	36	OFV91717.1	-			-		
Acidobacteria bacterium RIFCSPLOWO2_02_FULL_65_29	6	✓	39	OFW27858.1	✓	34	OFW27845.1	✓	33	OFW27859.1	-		
Acidobacteria bacterium RIFCSPLOWO2_02_FULL_67_36	6	✓	40	OFW11518.1	✓	38	OFW11459.1	✓	34	OFW11460.1	-		
Acidobacteria bacterium RIFCSPLOWO2_02_FULL_67_21	6	-			-			-			-		
Acidobacteria bacterium RIFCSPLOWO2_02_FULL_68_18	6	-			-			-			-		
Acidobacteria bacterium RIFCSPLOWO2_12_FULL_67_14b	6	✓	39	OFW46408.1	✓	37	OFW46352.1	✓	32	OFW46351.1	-		
Acidobacteria bacterium 13_1_20CM_2_65_9	6	-			-			-			-		
Acidobacteria bacterium 13_1_40CM_2_64_6	6	-			-			-			-		
Acidobacteria bacterium 13_1_40CM_65_14	6	✓	34	OLC40177.1	✓	36	OLC40174.1	✓	32	OLC40173.1	-		
Acidobacteria bacterium SCN 69-37	6	✓	42	ODS52724.1	✓	35	ODS52560.1	✓	31	ODS52559.1	-		
Acidobacteria bacterium Mor-1	22	-			-			-			✓		ANM31419.1
Acidobacteria bacterium RBG_13_68_16	23	-			-			-			-		
Acidobacteria bacterium 13_1_20CM_2_68_14	U	-			-			-			-		
Acidobacteria bacterium 13_1_40CM_2_68_10	U	-			-			-			-		
Acidobacteria bacterium 13_1_40CM_2_68_5	U	✓	44	OLD62390.1	✓	38	OLD62389.1	✓[b]		OLD62377.1	-		
Acidobacteria bacterium 13_1_40CM_4_69_4	U	-			-			-			-		

Key: SD = subdivision, ✓ = present, - = absent, SI = similarity (%) as blasted against the corresponding gene of *A. capsulatum* ATCC 51196 or *S. bohemicum* S15 (in case of the putative *fdpT*), AC = Accession number in NCBI database, U = unknown

[a] in these cases *hpnC* and *hpnD* are fused (see text); [b] partial gene

Table S4: Presence of the *shc*, *hpnH*, *hpnG*, and *hpnA* genes encoding enzymes in BHP synthesis in genomes of cultivated *Acidobacteria* based on BLAST searches of the protein sequence in the NCBI database.

***Acidobacteria*l genome**	**SD**	***shc***	**SI[a] (%)**	**AC**	***hpnH***	**SI[b] (%)**	**AC**	***hpnG***	**SI[b]**	**AC**	***hpnA***	**SI[b]**	**AC**
**Cultures**													
Acidobacterium capsulatum 161	1	✓	100	WP_015898362.1	✓	100	WP_015898363.1	✓	100	WP_015895985.1	✓	100	WP_015895986.1
Acidobacterium ailaaui PMMR2	1	✓	85	WP_026443152.1	-	-	-	✓	49	WP_026441645.1	✓	76	WP_026441644.1
Silvibacterium bohemicum S15	1	✓	82	WP_050061632.1	✓	92	WP_050061630.1	✓	54	WP_050062229.1	✓	81	WP_050062230.1
Acidobacteriaceae bacterium KBS 83	1	✓	81	WP_035222935.1	✓	82	WP_020710821.1	✓	39	WP_020710818.1	✓	76	WP_020710819.1
Acidobacteriaceae bacterium KBS 89	1	✓	78	WP_020713669.1	✓	81	WP_020713668.1	✓	39	WP_020713665.1	✓	74	WP_020713666.1
Terracidiphilus gabretensis S55	1	✓	78	WP_058185512.1	✓	71	WP_058185513.1	✓	40	WP_058185516.1	✓	71	WP_058185515.1
Edaphobacter aggregans Wbg-1	1	✓	76	WP_035349256.1	✓	87	WP_035349255.1	✓	45	WP_051978543.1	✓	76	WP_035349251.1
Acidobacteriaceae bacterium URHE0068	1	✓	78	WP_026448065.1	✓	70	WP_026448064.1	✓	40	WP_051566259.1	✓	71	WP_026448074.1
Acidobacteria bacterium KBS 146	1	✓	76	WP_026385916.1	✓	90	WP_026385917.1	✓	47	WP_051627860.1	✓	73	WP_026385919.1
Terriglobus saanensis SP1PR4	1	✓	75	WP_013568531.1	✓	82	WP_013568529.1	✓	37	WP_013568523.1	✓	74	WP_013568525.1
Granulicella mallensis MP5ACTX8	1	✓	74	WP_014264569.1	✓	86	WP_014264570.1	✓	39	WP_014264573.1	✓	74	WP_014264572.1
Granulicella tundricola MP5ACTX9	1	✓	71	WP_013579964.1	✓	82	WP_013579965.1	✓	40	WP_013579968.1	✓	75	WP_013579967.1
Granulicella pectinivorans TPB6011	1	✓	70<br>44	SFS15998.1<br>SFS15307.1	✓	86	SFS16003.1	✓	36	SFS16018.1	✓	74	SFS16012.1
Bryocella elongata SN10	1	✓	70	SEF69162.1	✓	85	SEF69136.1	✓	38	SEF69052.1	✓	69	SEF69081.1
Terriglobus roseus DSM 18391	1	✓	74	WP_014785831.1	✓	80	WP_014785830.1	✓	40	WP_014785827.1	✓	72	WP_014785828.1
Terriglobus sp. TAA 43	1	✓	70	WP_047488088.1	✓	81	WP_047488086.1	✓	40	WP_052200307.1	✓	71	WP_047488072.1
Terriglobus roseus GAS232	1	✓	71	SDF08232.1	✓	81	SDF08278.1	✓	38	SDF08494.1	✓	70	SDF08455.1
Terriglobus roseus AB35.6	1	✓	74	SEB38205.1	✓	80	SEB38193.1	✓	38	SEB38159.1	✓	72	SEB38171.1
Acidobacteriaceae bacterium TAA166	1	✓	71	WP_022845288.1	✓	88	WP_022845289.1	✓	45	WP_022845292.1	✓	71	WP_022845291.1
‘*Ca.* Koribacter versatilis Ellin345’	1	✓	68	ABF40741.1	✓	71	WP_011521820.1	✓	31	WP_011522545.1	✓	62	WP_011522544.1
Acidobacteriaceae bacterium KBS 96	3	✓	47	WP_020719493.1	✓	73	WP_020719381.1	✓	27	WP_020719491.1	✓	51	WP_020719492.1
‘*Ca.* Solibacter usitatus Ellin6076’	3	✓	44<br>46	WP_011683030.1<br>WP_011682956.1	✓	69	WP_011685281.1	✓	36	WP_011683032.1	✓	54	WP_011683031.1
Bryobacter aggregatus MPL3	3	✓	44	WP_035957816.1	✓	44	WP_051670105.1	✓	44	WP_031498836.1	✓	51	WP_031498835.1
Chloracidobacterium thermophilum B	4	✓	43	WP_014100779.1	✓	39	WP_014100993.1	✓	33	WP_014101332.1	✓	50	WP_014101352.1
Chloracidobacterium thermophilum OC10	4	✓	43	WP_058866228.1	✓	39	WP_058866121.1	✓	30	WP_058868517.1	✓	50	WP_058868498.1
Pyrinomonas methylaliphatogenes K22	4	-			-			-			-		
Luteitalea pratensis HEG_-6_39	6	-			-			-			-		
Holophaga foetida TMBS4	8	-			-			-			-		
Geothrix fermentans H-5	8	-			-			-			-		
Thermotomaculum hydrothermale AC55	10	-			-			-			-		
Thermoanaerobaculum aquaticum MP-01	23	-			-			-			-		
**Environmental genomes**													
Acidobacteriales bacterium 59-55	1	✓	76	OJV41826.1	✓	80	OJV41825.1	-			✓	80	OJV41823.1
Acidobacteriales bacterium 13_1_20CM_4_56_7	1	✓	68	OLE14569.1	✓	65	OLE14570.1	-			-		
Acidobacteriales bacterium 13_1_40CM_3_55_5	1	✓	68	OLD19462.1	-			✓	31	OLD19458.1	✓	64	OLD19459.1

Acidobacteriales bacterium 13_2_20CM_2_55_5	1	✓	68	OLB88617.1	✓	73	OLB88618.1	✓	31	OLB87675.1	✓	65	OLB87680.1
Acidobacteriales bacterium 13_2_20CM_55_8	1	✓	69	OLB21625.1	-			-			-		
Acidobacteria bacterium 13_1_20CM_2_60_10	2	✓	44	OLE83115.1	-			✓	25	OLE83117.1	✓	51	OLE83116.1
Acidobacteria bacterium 13_1_20CM_3_58_11	2	✓	44	OLE45836.1	-			-			-		
Acidobacteria bacterium 13_1_20CM_58_21	2	-			✓	74	OLD79942.1	✓	28	OLD83135.1	✓	52	OLD83134.1
Acidobacteria bacterium 13_1_40CM_4_57_6	2		45	OLC82649.1	-			-			✓	50	OLC82648.1
Acidobacteria bacterium 13_1_40CM_4_58_4	2	✓	45<br>43	OLC90948.1<br>OLC96281.1	✓	73<br>40	OLC89682.1<br>OLC90949.1	✓	24<br>24	OLC90951.1<br>OLC96285.1	✓	51<br>50	OLC96284.1<br>OLC90950.1
Acidobacteria bacterium 13_1_40CM_4_61_5	2	-			✓	75	OLC88768.1	✓	25	OLC88154.1	✓	50	OLC88155.1
Acidobacteria bacterium 13_2_20CM_2_57_6	2	✓	45	OLB87282.1	✓	74	OLB82578.1	✓	26	OLB88654.1	-		
Acidobacteria bacterium 13_2_20CM_57_17	2	✓	45	OLB40694.1	✓	74	OLB40168.1	✓	26	OLB40691.1	✓	50	OLB40692.1
Acidobacteria bacterium 13_2_20CM_57_7	2	✓	45	OLB21789.1	✓	74	OLB18125.1	✓	26	OLB21792.1	✓	50	OLB21791.1
Acidobacteria bacterium 13_2_20CM_58_27	2	✓	45	OLB28953.1	✓	73	OLB28086.1	✓	27	OLB28868.1	✓	50	OLB28869.1
Acidobacteria bacterium 13_1_40CM_56_16	2'	✓	44	OLC39085.1	✓	40	OLC39079.1	✓	23	OLC39077.1	✓	50	OLC39078.1
Acidobacteria bacterium RIFCSPLOWO2_02_FULL_59_13	1/3	-			-			✓	29	OFW14227.1	✓	53	OFW14226.1
Acidobacteria bacterium RIFCSPLOWO2_02_FULL_61_28	1/3	✓	51	OFV92199.1	✓	41	OFV92200.1	✓	32	OFV92202.1	✓	54	OFV92201.1
Acidobacteria bacterium RIFCSPLOWO2_12_FULL_54_10	1/3	-			✓	43	OFV98826.1	–			-		
Acidobacteria bacterium RIFCSPLOWO2_12_FULL_59_11	1/3	✓	47	OFW00329.1	✓	43	OFW00328.1	✓	28	OFW00326.1	✓	54	OFW00327.1
Acidobacteria bacterium RIFCSPLOWO2_12_FULL_60_22	1/3	-			-			✓	19	OFW36620.1	-		
Acidobacteria bacterium RIFCSPHIGHO2_02_FULL_67_57	3/4	-			-			-			-		
Acidobacteria bacterium RIFCSPHIGHO2_12_FULL_67_30	3/4	-			-			-			-		
Acidobacteria bacterium OLB17	4	-			-			-			-		
Acidobacteria bacterium 13_1_20CM_3_53_8	4	-			-			-			-		
Acidobacteria bacterium 13_1_40CM_3_55_6	4	-			-			-			-		
Acidobacteria bacterium RBG_16_70_10	4'	-			-			-			-		
Acidobacteria bacterium RIFCSPLOWO2_02_FULL_64_15	6	-			-			-			-		
Acidobacteria bacterium RIFCSPLOWO2_02_FULL_65_29	6	-			-			-			-		
Acidobacteria bacterium RIFCSPLOWO2_02_FULL_67_36	6	-			-			-			-		
Acidobacteria bacterium RIFCSPLOWO2_02_FULL_67_21	6	-			-			-			-		
Acidobacteria bacterium RIFCSPLOWO2_02_FULL_68_18	6	-			-			-			-		
Acidobacteria bacterium RIFCSPLOWO2_12_FULL_67_14b	6	-			-			-			-		
Acidobacteria bacterium 13_1_20CM_2_65_9	6	-			-			-			-		
Acidobacteria bacterium 13_1_40CM_2_64_6	6	-			-			-			-		
Acidobacteria bacterium 13_1_40CM_65_14	6	-			-			-			-		
Acidobacteria bacterium SCN 69-37	6	-			-			-			-		
Acidobacteria bacterium Mor-1	22	✓	47	ANM31425.1	✓	39	ANM31424.1	-			?	28	ANM30436.1
Acidobacteria bacterium RBG_13_68_16	23	-			-			-			-		
Acidobacteria bacterium 13_1_20CM_2_68_14	U	-			-			-			-		
Acidobacteria bacterium 13_1_40CM_2_68_10	U	-			-			-			-		
Acidobacteria bacterium 13_1_40CM_2_68_5	U	-			-			-			-		
Acidobacteria bacterium 13_1_40CM_4_69_4	U	-			-			-			-		

Key: SD = subdivision, ✓ = present, - = absent, SI = similarity (%) as blasted against the corresponding gene of *A. capsulatum* ATCC 51196, AC = Accession number in NCBI database, U = unknown

Table S5: Presence of the *hpnB, hpnI, hpnJ* and *hpnK* genes encoding enzymes in BHP synthesis in genomes of cultivated *Acidobacteria* and *Acidobacterial* metagenomes based on BLAST searches of the protein sequence in the NCBI database.

***Acidobacteria*l (meta)genome**	**SD**	***hpnB***	**SI (%)[b]**	**AC**	***hpnI***	**SI (%)[b]**	**AC**	***hpnJ***	**SI (%)[b]**	**AC**	***hpnK***	**SI[b]**	**AC**
**Cultures**													
Acidobacterium capsulatum ATCC 51196	1	-			✓	100	WP_015897110.1	✓	100	WP_015898078.1	-		
Acidobacterium ailaaui PMMR2	1	-			✓	60	WP_026442134.1	✓	91	WP_026441760.1	-		
Silvibacterium bohemicum S15	1	-			✓	59	WP_050060510.1	✓	90	WP_050062222.1	-		
Acidobacteriaceae bacterium KBS 83	1	-			✓	58	WP_035223311.1	✓	86	WP_020710808.1	-		
Acidobacteriaceae bacterium KBS 89	1	-			✓	57	WP_020715618.1	✓	84	WP_020713421.1	-		
Terracidiphilus gabretensis S55	1	-			✓	56	WP_058187425.1	✓	83	WP_058185522.1	-		
Edaphobacter aggregans DSM 19364	1	✓	90	WP_035352220.1	✓	56	WP_064742595.1	✓	87	WP_035350369.1	-		
Acidobacteriaceae bacterium URHE0068	1	✓	57	WP_026446012.1	✓	55	WP_026448633.1	✓	83	WP_026448084.1	-		
Acidobacteria bacterium KBS 146	1	-			✓	56	WP_035176462.1	✓	87	WP_026386227.1	-		
Terriglobus saanensis SP1PR4	1	-			✓	54	WP_049781167.1	✓	85	WP_013568400.1	-		
Granulicella mallensis MP5ACTX8	1	✓	100	WP_014265833.1	✓	55	WP_014263407.1	✓	84	WP_014265795.1	-		
Granulicella tundricola MP5ACTX9	1	-			✓	54	WP_013578476.1	✓	82	WP_013580018.1	-		
Granulicella pectinivorans DSM 21001	1	-			✓	55	SFS03073.1	✓	85	SFS16903.1	-		
Bryocella elongata DSM 22489	1	-			✓	54	SEG20810.1	✓	85	SEF64546.1	-		
Terriglobus roseus DSM 18391	1	-			✓	52	WP_014784111.1	✓	81	WP_014785733.1	-		
Terriglobus sp. TAA 43	1	✓	57	WP_047494619.1	✓	50	WP_047492068.1	✓	83	WP_047487014.1	-		
Terriglobus roseus GAS232	1	✓	56	SDF88027.1	✓	51	SDF78336.1	✓	83	SDF24644.1	-		
Terriglobus roseus AB35.6	1	-			✓	54	SEB85419.1	✓	82	SEB36957.1	-		
Acidobacteriaceae bacterium TAA166	1	-			✓	54	WP_022843429.1	✓	85	WP_022844919.1	-		
'*Ca.* Koribacter versatilis Ellin345'	1	-			✓	49	WP_011521530.1	✓	76	WP_011523121.1	-		
Acidobacteriaceae bacterium KBS 96	3	✓	46	WP_020722380.1	✓	38	WP_020720612.1	✓	65	WP_020720609.1	-		
'*Ca.* Solibacter usitatus Ellin6076'	3	✓	44	WP_011685564.1	✓	40	WP_049873324.1	✓	68	WP_011689127.1	-		
Bryobacter aggregatus MPL3	3	-			✓	39	WP_051669583.1	✓	63	WP_031496923.1	-		
Chloracidobacterium thermophilum B	4	-			✓	39	WP_014100987.1	✓	64	WP_014100991.1	✓	100	WP_014100990.1
Chloracidobacterium thermophilum	4	-			✓	42	WP_058866115.1	✓	64	WP_058866119.1	✓	98	WP_058866118.1
Pyrinomonas methylaliphatogenes K22	4	-			-			-			-		
Luteitalea pratensis HEG_-6_39	6	-			-			-			-		
Holophaga foetida DSM 6591	8	-			-			-			-		
Geothrix fermentans DSM 14018	8	-			-			-			-		
Thermotomaculum hydrothermale AC55	10	-			-			-			-		
Thermoanaerobaculum aquaticum MP-01	23	-			-			-			-		
**Environmental genomes**													
Acidobacteria bacterium 59-55	1	-			✓	56	OJV44113.1	✓	82	OJV41462.1	-		
Acidobacteriales bacterium 13_1_20CM_4_56_7	1	-			-			-			-		
Acidobacteriales bacterium 13_1_40CM_3_55_5	1	✓	42	OLD15195.1	-			✓	76	OLD18777.1	-		
Acidobacteriales bacterium 13_2_20CM_2_55_5	1	-			✓	48	OLB84463.1	✓	73	OLB87669.1	-		
Acidobacteriales bacterium 13_2_20CM_55_8	1	-			✓	49	OLB20242.1	✓	77	OLB22759.1	-		

Acidobacteria bacterium 13_1_20CM_2_60_10	2	-			-			-			-		
Acidobacteria bacterium 13_1_20CM_3_58_11	2	✓	37	OLE47118.1	-			-			-		
Acidobacteria bacterium 13_1_20CM_58_21	2	✓	38	OLD82715.1	-			-			-		
Acidobacteria bacterium 13_1_40CM_4_57_6	2	✓	40	OLC84446.1	✓	39<br>34	OLC83552.1<br>OLC83269.1	✓	64	OLC83549.1	-		
Acidobacteria bacterium 13_1_40CM_4_58_4	2	✓	39	OLC96924.1	✓	40	OLC91614.1	✓	62	OLC91617.1	-		
Acidobacteria bacterium 13_1_40CM_4_61_5	2	✓	43	OLC88360.1	-			-			-		
Acidobacteria bacterium 13_2_20CM_2_57_6	2	✓	42	OLB84357.1	-			-			-		
Acidobacteria bacterium 13_2_20CM_57_17	2	✓	40	OLB34865.1	✓	39	OLB34984.1	✓	63	OLB34980.1	-		
Acidobacteria bacterium 13_2_20CM_57_7	2	-			✓	40	OLB18610.1	✓	63	OLB18614.1	-		
Acidobacteria bacterium 13_2_20CM_58_27	2	✓	39	OLB32266.1	✓	40	OLB30651.1	✓	65	OLB30648.1	-		
Acidobacteria bacterium 13_1_40CM_56_16	2’	-			✓	44<br>40<br>34	OLC31961.1<br>OLC29808.1<br>OLC37113.1	✓	62	OLC31958.1	-		
Acidobacteria bacterium RIFCSPLOWO2_12_FULL_59_13	1/3	-			✓	40	OFW16935.1	✓	66	OFW16931.1	-		
Acidobacteria bacterium RIFCSPLOWO2_02_FULL_61_28	1/3	-			✓	38<br>35	OFV99257.1<br>OFV98740.1	✓	63	OFV99253.1	-		
Acidobacteria bacterium RIFCSPLOWO2_12_FULL_54_10	1/3	-			-			✓	65	OFV93540.1	-		
Acidobacteria bacterium RIFCSPLOWO2_12_FULL_59_11	1/3	-			-			-			-		
Acidobacteria bacterium RIFCSPLOWO2_12_FULL_60_22	1/3	-			✓	34	OFW35345.1	-			-		
Acidobacteria bacterium RIFCSPHIGHO2_02_FULL_67_57	3/4	-			-			-			-		
Acidobacteria bacterium RIFCSPHIGHO2_12_FULL_67_30	3/4	-			-			-			-		
Acidobacteria bacterium OLB17	4	-			-			-			-		
Acidobacteria bacterium 13_1_20CM_3_53_8	4	-			-			-			-		
Acidobacteria bacterium RBG_16_70_10	4’	-			-			-			-		
Acidobacteria bacterium RIFCSPLOWO2_02_FULL_64_15	6	-			-			-			-		
Acidobacteria bacterium RIFCSPLOWO2_02_FULL_65_29	6	-			-			-			-		
Acidobacteria bacterium RIFCSPLOWO2_02_FULL_67_36	6	-			-			-			-		
Acidobacteria bacterium RIFCSPLOWO2_02_FULL_67_21	6	-			-			-			-		
Acidobacteria bacterium RIFCSPLOWO2_02_FULL_68_18	6	-			-			-			-		
Acidobacteria bacterium RIFCSPLOWO2_12_FULL_67_14b	6	-			-			-			-		
Acidobacteria bacterium 13_1_20CM_2_65_9	6	-			-			-			-		
Acidobacteria bacterium 13_1_40CM_2_64_6	6	-			-			-			-		
Acidobacteria bacterium 13_1_40CM_65_14	6	-			-			-			-		
Acidobacteria bacterium SCN 69-37	6	-			-			-			-		
Acidobacteria bacterium Mor-1	22	✓	39	ANM31838.1	-			-					
Acidobacteria bacterium RBG_13_68_16	23	-			-			-			-		
Acidobacteria bacterium 13_1_20CM_2_68_14	U	-			-			-			-		
Acidobacteria bacterium 13_1_40CM_2_68_10	U	-			-			-			-		
Acidobacteria bacterium 13_1_40CM_2_68_5	U	-			-			-			-		
Acidobacteria bacterium 13_1_40CM_4_69_4	U	-			-			-			-		

Key: SD = subdivision, ✓ = present, - = absent, SI = similarity (%) as blasted against the corresponding gene of *A. capsulatum* ATCC 51196, *G. mallensis* MP5ACTX8 or *C. thermophilum* B, AC = Accession number in NCBI database, U = unknown

Table S6: Presence of the *hpnO* gene encoding an enzyme in BHP synthesis in genomes of cultivated *Acidobacteria* and *Acidobacterial* metagenomes based on BLAST searches of the protein sequence in the NCBI database.

***Acidobacteria*l genomes**	**SD**	***hpnO***	**SI (%)[b]**	**AC**
**Cultures**				
Acidobacterium capsulatum 161	1	✓	100	WP_015897314.1
Acidobacterium ailaaui PMMR2	1	✓	79	WP_044933846.1
Silvibacterium bohemicum S15	1	✓	82	WP_050058300.1
Acidobacteriaceae bacterium KBS 83	1	✓	82	WP_020707688.1
Acidobacteriaceae bacterium KBS 89	1	✓	77	WP_051098220.1
Terracidiphilus gabretensis S55	1	✓	75	WP_058188695.1
Edaphobacter aggregans Wbg-1	1	✓	78	
Acidobacteriaceae bacterium URHE0068	1	✓	79	WP_035237387.1
Acidobacteria bacterium KBS 146	1	✓	77	WP_026387216.1
Terriglobus saanensis SP1PR4	1	-		
Granulicella mallensis MP5ACTX8	1	✓	77	WP_044178275.1
Granulicella tundricola MP5ACTX9	1	-		
Granulicella pectinivorans TPB6011	1	-		
Bryocella elongata SN10	1	-		
Terriglobus roseus DSM 18391	1	-		
Terriglobus sp. TAA 43	1	-		
Terriglobus roseus GAS232	1	-		
Terriglobus roseus AB35.6	1	-		
Acidobacteriaceae bacterium TAA166	1	-		
'*Ca.* Koribacter versatilis Ellin345'	1	✓	72	WP_011524788.1
Acidobacteriaceae bacterium KBS 96	3	✓	60	WP_044952283.1
'*Ca.* Solibacter usitatus Ellin6076'	3	✓	56	WP_011684194.1
Bryobacter aggregatus MPL3	3	✓	54	WP_035957839.1
Chloracidobacterium thermophilum B	4	✓	53	WP_014098758.1
Chloracidobacterium thermophilum OC1	4	-		
Pyrinomonas methylaliphatogenes K22	4	✓	58	WP_041978156.1
Luteitalea pratensis HEG_-6_39	6	✓	57	AMY11128.1
Holophaga foetida TMBS4	8	-		
Geothrix fermentans H-5	8	-		
Thermotomaculum hydrothermale AC55	10	-		
Thermoanaerobaculum aquaticum MP-01	23	-		
**Environmental genomes**				
Acidobacteriales bacterium 59-55	1	-		
Acidobacteriales bacterium 13_1_20CM_4_56_7	1	✓	72	OLE11736.1
Acidobacteriales bacterium 13_1_40CM_3_55_5	1	-		
Acidobacteriales bacterium 13_2_20CM_2_55_5	1	-		
Acidobacteriales bacterium 13_2_20CM_55_8	1	-		
Acidobacteria bacterium 13_1_20CM_2_60_10	2	✓	61	OLE86312.1

Acidobacteria bacterium 13_1_20CM_3_58_11	2	✓	58	OLE46603.1
Acidobacteria bacterium 13_1_20CM_58_21	2	-		
Acidobacteria bacterium 13_1_20CM_4_57_6	2	-		
Acidobacteria bacterium 13_1_40CM_4_58_4	2	-		
Acidobacteria bacterium 13_1_40CM_4_61_5	2	-		
Acidobacteria bacterium 13_2_20CM_2_57_6	2	-	59	OLB88366.1
Acidobacteria bacterium 13_2_20CM_57_17	2	-		
Acidobacteria bacterium 13_2_20CM_57_7	2	✓	59	OLB17291.1
Acidobacteria bacterium 13_2_20CM_58_27	2	✓	56	OLB27672.1
Acidobacteria bacterium 13_1_20CM_56_16	2'	✓	56	OLC29154.1
Acidobacteria bacterium RIFCSPLOWO2_02_FULL_59_13	1/3	✓	57	OFW10540.1
Acidobacteria bacterium RIFCSPLOWO2_02_FULL_61_28	1/3	✓	60	OFV98128.1
Acidobacteria bacterium RIFCSPLOWO2_12_FULL_54_10	1/3	-		
Acidobacteria bacterium RIFCSPLOWO2_12_FULL_59_11	1/3	✓	60	OFW06698.1
Acidobacteria bacterium RIFCSPLOWO2_12_FULL_60_22	1/3	✓	57	OFW10540.1
Acidobacteria bacterium RIFCSPHIGHO2_02_FULL_67_57	3/4	-		
Acidobacteria bacterium RIFCSPHIGHO2_12_FULL_67_30	3/4	-		
Acidobacteria bacterium OLB17	4	✓	49	KXK05977.1
Acidobacteria bacterium 13_1_20CM_3_53_8	4	✓	55	OLE53531.1
Acidobacteria bacterium RBG_16_70_10	4'	-		
Acidobacteria bacterium RIFCSPLOWO2_02_FULL_64_15	6	-		
Acidobacteria bacterium RIFCSPLOWO2_02_FULL_65_29	6	✓	56	OFW28539.1
Acidobacteria bacterium RIFCSPLOWO2_02_FULL_67_36	6	✓	56	OFW09896.1
Acidobacteria bacterium RIFCSPLOWO2_02_FULL_67_21	6	✓	57	OFW14803.1
Acidobacteria bacterium RIFCSPLOWO2_02_FULL_68_18	6	-		
Acidobacteria bacterium RIFCSPLOWO2_12_FULL_67_14b	6	✓	53	OFW42207.1
Acidobacteria bacterium 13_1_20CM_2_65_9	6	-		
Acidobacteria bacterium 13_1_40CM_2_64_6	6	-		
Acidobacteria bacterium 13_1_40CM_65_14	6	-		
Acidobacteria bacterium SCN 69-37	6	-		
Acidobacteria bacterium Mor-1	22	-		
Acidobacteria bacterium RBG_13_68_16	23	-		
Acidobacteria bacterium 13_1_20CM_2_68_14	U	-		
Acidobacteria bacterium 13_1_40CM_2_68_10	U	✓	50	OLD67358.1
Acidobacteria bacterium 13_1_40CM_2_68_5	U	✓	50	OLD62359.1
Acidobacteria bacterium 13_1_40CM_4_69_4	U	-		

Key: SD = subdivision, ✓ = present, - = absent, SI = similarity (%) as blasted against the corresponding gene of *A. capsulatum* ATCC 51196, AC = Accession number in NCBI database, U = unknown
